# Longitudinal Analysis of the Microbiota Composition and Enterotypes of Pigs from Post-Weaning to Finishing

**DOI:** 10.3390/microorganisms7120622

**Published:** 2019-11-28

**Authors:** Mathilde Le Sciellour, David Renaudeau, Olivier Zemb

**Affiliations:** 1 UMR 1348 PEGASE, Agrocampus-Ouest, INRA, F-35590 Saint-Gilles, Francedavid.renaudeau@inra.fr (D.R.); 2 UMR 1388 GenPhySE, Université de Toulouse, INRA, INPT, ENVT, F-31320 Castanet Tolosan, France

**Keywords:** longitudinal analysis, microbiota, finishing pig, enterotype

## Abstract

The present study aimed at investigating the evolution of pigs’ fecal microbiota composition from post-weaning to finishing in a longitudinal analysis. The experiment was conducted on 160 Pietrain × (Large White × Landrace) castrated male and female pigs in two replicates. Feces were collected at 52, 99, 119, 140, and 154 days of age for further 16S rRNA sequencing to analyze the microbiota composition. Pig microbiota evolved strongly from 52 to 99 days of age with an increased abundance of Streptococcaceae and a decreased abundance of Lactobacillaceae. During the finishing stage, microbiota kept evolving at a slower rate. To link the community structure to the performances, the fecal samples were clustered into enterotypes sharing a similar bacterial composition. At 52 days, two enterotypes dominated either by *Lactobacillus* or by *Prevotella*–*Sarcina* were identified. They differed from the two enterotypes determined from 99 to 154 days which were dominated either by *Lactobacillus* or by *Turicibacter*–*Clostridium* *sensu* *stricto*. During this time period, 75% of the pigs switched enterotypes. The enterotypes were not related to differences in the overall growth or feeding performance. The enterotype definition was time-dependent and seemed to be related to the sex type at 99 days of age.

## 1. Introduction

Pigs’ gut microbiota is composed of more than 700 metagenomic species [1]. Gut microbiota plays a central role in its host’s metabolism and health by participating in nutrient and fat metabolism and absorption, by breaking down toxins, by being a key component of the pig immune system, and by interacting with the peripheral nervous system. Particularly, the gut microbiota helps in degrading indigestible carbohydrates contained in fibers [2,3]. In turn, fibers can also impact the relative abundance of the microbial species [4,5,6,7]. Interestingly, pigs are exposed to various diets that are increasingly rich in fiber from weaning to finishing stage, and their microbiota evolves continuously [8]. However, the microbiota composition also evolves during the finishing stage even when the diet is constant [8], so that the diet and the age of the animal both impact the microbial equilibrium.

Recent advances underlined that several equilibrium states—i.e., enterotypes—are possible. Thus, humans are divided into three enterotypes [9], with functional implications such as dietary metabolism [10]. The same methodology applied on pigs between 14 and 70 days of age generated two enterotypes [11,12] and retrieved the co-exclusion between *Prevotella* and *Ruminococcus* observed in humans [9], suggesting some common functional architecture of the pig and human microbiota. However, the relationship between enterotypes and pigs’ performance is unclear. The *Ruminococcus*-dominated enterotype presented a higher average daily gain (ADG) from 22 to 28 days of age (during lactation) but lower ADG from 29 to 70 days of age [11]. However, the enterotypes established on 1200 pigs at 118 and 196 days of age were not related with ADG [13]. This study also demonstrated that the enterotypes strongly depended on the age. The longitudinal analysis in our experiment aimed at describing the enterotypes from post-weaning to finishing in 52, 99, 119, 140, and 154 days old pigs.

## 2. Materials and Methods

The experiment has been authorized by the French legislation on animal experimentation and ethics (authorization: 2016022415253973, approved on the 7 April 2017).

### 2.1. Animals and Experimental Design

The present study was based on an existing experiment where the animals had been exposed to a diet naturally contaminated with deoxynivalenol (DON). We choose to check afterward that the DON-contaminated diet did not affect the conclusions of the present study on the microbiota classification. The study was conducted on 160 Pietrain × (Large White × Landrace) castrated male and female pigs. Pigs were raised in two replicates from April to October 2017 in the INRA experimental facilities at the Unité Expérimentale Porcs de Rennes located in Saint Gilles, France. A commercial grower feed was provided ad libitum to the pigs from 52 to 91 days of age (Table S1). A transition to a finisher feed was applied between 91 and 98 days of age. The pigs were divided into four experimental groups. A control group (CC) received ad libitum a control finisher feed formulated based on 75% corn and 18% soybean meal (10.7 MJ/kg net energy) from 99 to 154 days of age (Table S1). A DON-contaminated diet was formulated similarly with a naturally-contaminated corn containing 4.8 mg DON/kg. The experimental groups were submitted to a 7-day DON challenge on day 113 (DC group), day 134 (CD group) and on both days 113 and 134 (DD group) (Figure S1). During the pre- and post-challenge periods, pigs received the control finisher feed. During the whole experiment, pigs had free access to water.

### 2.2. Measurements

The live body weight (BW) and feed intake were automatically recorded daily for each individual pig. The average daily gain (ADG), the average daily feed intake (ADFI), and the feed conversion ratio (FCR = ADFI/ADG) were calculated between 99 and 113 days, 113 and 119 days, 119 and 134 days, 134 and 140 days, 140 and 154 days, and during the whole finishing stage between 99 and 154 days of age.

In both replicates, the fecal samples from all pigs were collected at 99 and 154 days of age. Due to the time constraint, only females were sampled at 119 and at 140 days of age. In the second replicate, additional fecal samples were also collected from all pigs at 52 days of age. Feces were collected directly from the rectum, stored in barcoded tubes, immediately snap-frozen in liquid nitrogen and stored at −80 °C until DNA extraction.

### 2.3. DNA Sequencing

The DNA was extracted from 50 mg of feces using the ZR-96 Soil Microbe DNA kit (Zymo Research, Irvine, CA, USA) according to the manufacturer’s instructions [14]. A PCR was applied to amplify the V3-V4 region of the 16S rRNA gene using F460 and R460 primers (F460: CTTTCCCTACACGACGCTCTTCCGATCTACGGRAGGCAGCAG, R460: GGAGTTCAGACGTGTGCTCTTCCGATCTTACCAGGGTATCTAATCCT). The PCR was run in 30 cycles with an annealing temperature of 65 °C. Sequencing was performed with Illumina MiSeq V3 chemistry and the 2 × 250 bp paired-end sequences were cleaned internally for length, and undetermined nucleotides. These sequencing and cleaning steps were performed at the GeT-PlaGE platform (Toulouse, France).

VSEARCH software [15] was used to remove chimeras from the sequences. From the sequences, a de novo clustering resulted into operational taxonomic units (OTU) with a threshold of 0.97 similarity. The abundance of each OTU in each sample was recorded. Usearch software (9.2.64_i86linux32 version) was used to assign each OTU to a taxonomic affiliation based on the SILVA database (version 123, 97% similarity threshold) [16]. PICRUST version 2.0.3-b was applied to convert the OTU table to a table of abundance of MetaCyc pathways. The pathways that were different between at least one group and another were identified with a non-parametric Kruskal test adjusted for the false discovery rate by the Benjamini–Hochberg method. The Conover posthoc test was applied to quantify the differences between the two enterotypes at the post-weaning stage and the two enterotypes at the fattening stage.

### 2.4. Calculations and Statistical Analysis

The R 3.5.1. software [17] was used to run the statistical analyses. Based on the phyloseq package [18] and an OTU table rarefied at 15,752 sequences, the number of OTU and the Shannon index described the microbiota diversity. Due to the lack of fecal samples at 119 and 140 days of age in male pigs, we chose to analyze the age impact on the microbiota composition in females only. The diversity indexes (number of OTU and Shannon index) and the phyla relative abundance were compared between ages using Kruskal–Wallis tests. To visualize the evolution of the main genera abundance through time, we used a local polynomial regression fitting (degree 2) as proposed by the SplinectomeR package [19].

A non-metric distance scaling (NMDS) plot was used to represent the similarities between samples based on the Bray–Curtis distance matrix. A pairwise MANOVA was applied on this distance matrix to evaluate the impact of age. The bacterial community was considered significantly different between groups if the adjusted false discovery rate (FDR) was under 0.05 after 999 permutations.

To identify samples with similar bacterial composition, enterotypes at 52, 99, 119, 140, and 154 days were constituted following the methodology from Arumugam et al. [9]. Briefly, from the relative genus abundance, a Jensen–Shannon divergence matrix was calculated. The partitioning around medoids clustering algorithm was applied and the optimal number of clusters (corresponding to enterotypes) was assessed using the Calinski–Harabasz index. The silhouette coefficient was used to evaluate the significance of this clustering. Kruskal–Wallis tests compared the genus relative abundance between the resulting enterotypes at each age. Chi-squared tests were used to analyze the repartition of the sex types, or the DON-challenged groups among the enterotypes. The effects of the enterotypes at each age on pigs’ performance were analyzed using ANOVA models. The sex, the replicate, the DON challenge, the body weight at 99 days of age, and their interactions were included in the models.

## 3. Results

Live BW increased from 55 ± 5 kg at 99 days of age to 110 ± 11 kg at 154 days. The ADFI increased from 2.4 ± 0.4 kg/day to 3.3 ± 0.5 kg/day between the beginning and the end of the trial. Due to illness, some animals had to be removed from the experiment. Furthermore, some fecal samples could not be collected from some animals at one time point. At the end of the experiment, 513 fecal samples from 143 pigs had been sequenced for microbiota analysis (Figure S2). The number of sequences ranged from 15,752 to 80,731 (21,156 ± 12,226 sequences on average). All the samples were kept for further statistical analysis. After filtration for the rare OTU under 0.01% relative abundance, 1556 OTU remained.

According to Wilcoxon tests applied on control and challenged female pigs at 119 and at 140 days of age, the DON challenge did not significantly impact the number of OTU, the Shannon index, or the relative abundance of the main phyla (*p* > 0.05) (Table S2). Following this result, we chose to group all female pigs (control and challenged) to evaluate the impact of the age on microbiota composition. The richness and Shannon index of the fecal microbial community increased between post-weaning and finishing stages (*p* < 0.05), and remained stable during the finishing stage from 99 to 154 days (Table 1).

**Figure 1:**
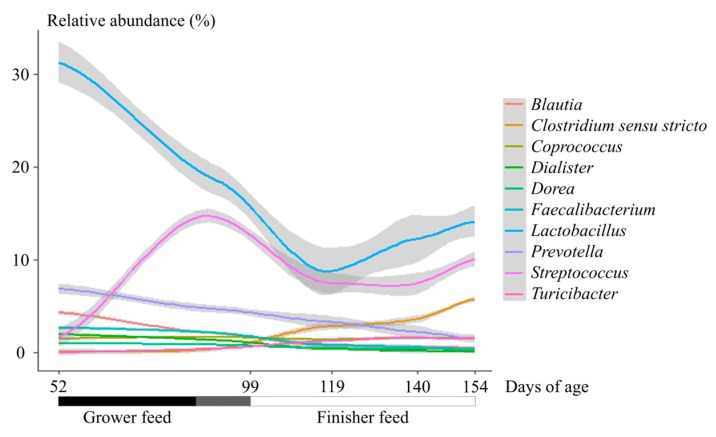
Evolution of the relative abundance of 10 main genera across ages in female pigs.

The relative abundance of Firmicutes increased between 52 and 99 days (*p* < 0.05), and a stability was achieved from day 99. The relative abundance of Bacteroidetes decreased all over the trial (*p* < 0.05). The relative abundance of Spirochaetes increased and the relative abundance of Actinobacteria decreased during the finishing stage (*p* < 0.05). The main represented family among the samples was Lactobacillaceae. The abundance of Lactobacillaceae (mainly *Lactobacillus*), Lachnospiraceae, Ruminococcaceae and Veillonellaceae decreased between 52 to 119 days, and were stable afterward. The abundance of Prevotellaceae (mainly *Prevotella*) decreased between 52 and 99 days, was stable between 99 and 140 days, and decreased at the end of the trial. The abundance of Streptococcaceae (mainly *Streptococcus*) increased between 52 and 99 days, decreased between 99 and 119 days, and was stable afterward. The *Clostridium sensu stricto* relative abundance increased between 52 and 99 days, was stable between 99 and 140 days, and increased at the end of the trial, in contrast with the *Prevotella* relative abundance (Figure 1). Based on the control samples, the structure of the bacterial community evolved across ages but the dispersion at each age remained homogenous (*p* = 0.09). The NMDS grouped the samples according to the age (Figure S3) and this separation was confirmed using the MANOVA (FDR < 0.05 for each pairwise comparison).

**Figure 2:**
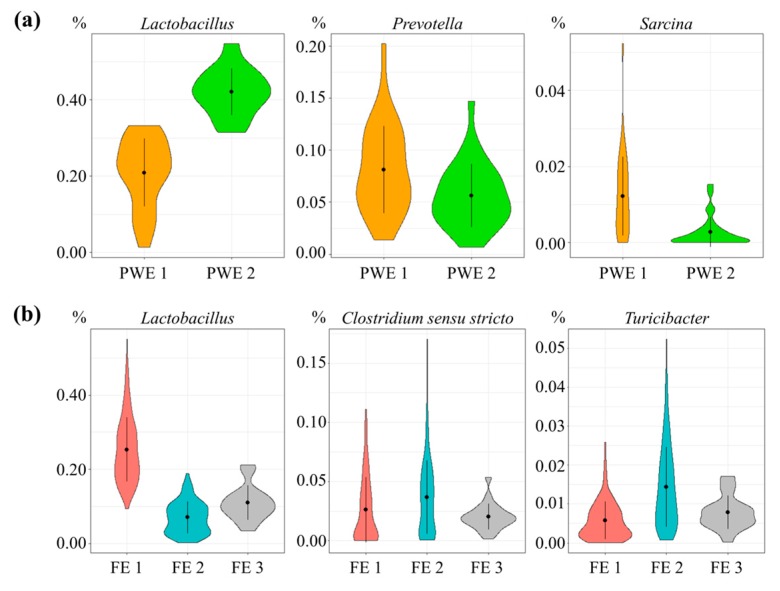
Relative abundance (%) of the three main genera contributing (**a**) to the two enterotypes in post-weaning enterotype 1 (PWE1) (in orange) and enterotype 2 (PWE2) (in green) and (**b**) to the three enterotypes in finishing enterotype 1 (FE1) (in red), finishing enterotype 1 (FE2) (in turquoise), and finishing enterotype 1 (FE3) (in grey).

Microbiota composition clustered into two enterotypes at 52 days of age, named post-weaning enterotype 1 (PWE1) and post-weaning enterotype 2 (PWE2) (Figure 2a and Figure S4). The microbial diversity in PWE1 was lower compared to PWE2 according to the number of OTU (2605 ± 313 vs. 2953 ± 258, *p* < 0.001), as was the Shannon index (6.60 ± 0.19 vs. 6.76 ± 0.16, *p* < 0.05). The PWE1 enterotype was dominated by *Prevotella*, *Sarcina*, *Treponema*, *Dialister*, *Mitsuokella*, *Clostridium sensu stricto*, *Fusicatenibacter*, *Roseburia*, *Blautia*, *Eubacterium*, *Enterococcus*, and *Desulfovibrio* (*p* < 0.05), whereas *Lactobacillus* and *Megasphaera* were more abundant in the PWE2 enterotype (Table S3). The ADG, ADFI, and FCR between 99 and 154 days of age did not differ between the enterotypes at 52 days.

Microbiota composition of finishing pigs clustered into two enterotypes at 99, 140, and 154 days of age, referred as finishing enterotype 1 (FE1) and finishing enterotype 2 (FE2) (Figure 2b and Figure S4). The microbial diversity in FE1 was greater compared to FE2 according to the number of OTU (3786 ± 320 vs. 3611 ± 303, *p* < 0.001) but the Shannon index did not differ (7.16 ± 0.13 vs. 7.13 ± 0.14, *p* = 0.73). In the FE1 enterotype, *Lactobacillus* and *Erysipelotrichaceae incertae sedis* were more abundant, whereas the FE2 enterotype was dominated by *Turicibacter*, *Clostridium sensu stricto*, *Sarcina*, *Clostridium XI*, *Treponema*, *Fibrobacter*, *Corynebacterium*, *Staphylococcus*, and *Clostridium IV* (*p* < 0.05).

The ADG, ADFI, and FCR did not differ between the enterotypes FE1 and FE2 (Table 2). At 119 days of age, a third enterotype named FE3 was observed with intermediate genera relative abundance between FE1 and FE2 (Figure 2b and Figure S4). Pigs in FE3 had greater ADG between 113 and 119 days of age compared to pigs in FE2. However, no significant difference was observed later in the experiment or between FE3 and FE1 (Table 2). The four experimental groups (CC, DC, CD, and DD) were equally represented in FE1, in FE2, and in FE3 (*p* = 0.50 in a Chi-squared test).

**Figure 3:**
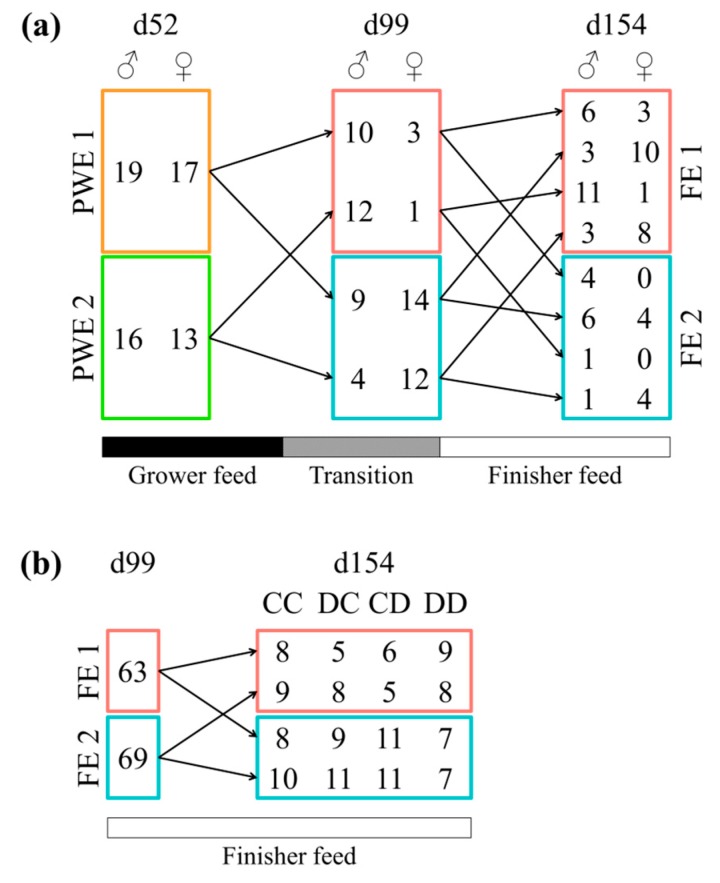
Number of pigs switching between enterotypes according to (**a**) the sex type (castrated males and females) or (**b**) the exposition to mycotoxins via the diet. Enterotypes from the post-weaning stage are represented by PWE1 and PWE2 and from the finishing stage by FE1 and FE2. Figure (**a**) was based on castrated males and females from the second replicate only. In Figure (**b**), based on castrated and females from both replicates, the CC, DC, CD, and DD experimental groups correspond to pigs fed with a control finisher diet, exposed to a DON-contaminated diet between 113 d and 119 d, between 134 d et 140 d, and between both 113 d and 119 d and 134 d and 140 d of age, respectively.

**Figure 4:**
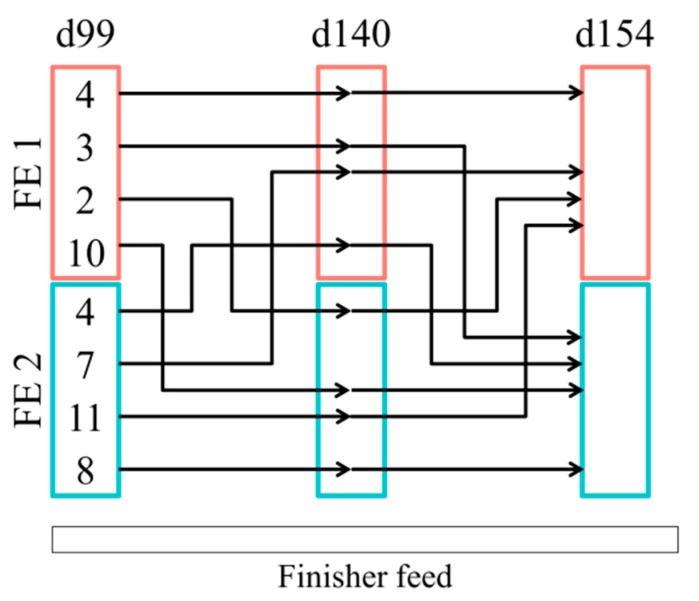
Number of female pigs switching between enterotypes (FE1 and FE2) during the finishing stage (between 99 and 154 d of age).

A total of 13 pigs moved from PWE1 enterotype to FE1, 23 from PWE1 to FE2, 13 from PWE2 to FE1, and 16 from PWE2 to FE2 (Figure 3a). In post-weaning, castrated males and females were equally represented in the enterotypes (*p* = 1.00 in a Chi-squared test). At 99 days of age, sex impacted the enterotype, with the females more represented in the FE2 compared to the FE1 (*p* < 0.001). However, at 154 days of age, the enterotype was independent of the sex (*p* = 0.69). During the finishing stage, the instability of the enterotypes observed in pigs switching enterotypes (in comparison with the stability when pigs remain clustered in the same enterotype all experiment long) tended to depend on the sex (*p* = 0.08) (Figure 3a). Furthermore, a switch between the enterotypes during the finishing stage was independent of an exposure to DON (*p* = 1.00) (Figure 3b). At 154 days of age, pigs receiving the DON-contaminated diet were equally represented in each enterotype (*p* = 0.32) (Figure S5). More precisely, when looking specifically at the 49 females pigs between 99 and 154 days, four pigs remained in the FE1 enterotype and eight in the FE2 enterotype (Figure 4). The other pigs moved from one enterotype to another enterotype once or twice during this time period.

## 4. Discussion

This study focused on the evolution of pigs’ fecal microbiota composition from post-weaning to finishing at both bacteria and ecosystem levels. In our experiment, the diversity increased when the pigs were aging, as previously observed [20,21,22]. At the phylum level, the Firmicutes’ relative abundance increased especially between post-weaning and finishing periods [22] and remained stable during the late-stages [23]. In accordance with Han et al. [23], the relative abundance of Spirochaetes increased during the finishing period. However, our results on the relative abundances of Spirochaetes, Actinobacteria and Bacteroidetes differed from those of Zhao et al. [22] where Actinobacteria gradually replaced Spirochaetes and Bacteroidetes increased as the pigs grow older. At a family level, the main changes in abundance appeared between post-weaning and finishing in accordance with previous findings [21]. Furthermore, we demonstrated that the microbiota composition kept evolving during all tested stages based on the NMDS and the relative abundance at family and genus levels in line with the results of Kim et al. [24] despite previous studies demonstrating that a stabilized composition of the microbiota was reached around three months of age [22,23].

Based on our results, the enterotype definition was age-dependent, as two enterotypes (PWE1 and PWE2) were identified at 52 days of age and another two (FE1 and FE2) were identified between 99 and 154 days. The constant evolution of the microbiota composition before three months of age [22,23] might explain that different enterotypes can be observed at different time points. Our PWE1 and PWE2 enterotypes described at 52 days of age differed from the enterotypes previously described at 60 days of age [12]: the PEA enterotype dominated by *Ruminococcus* and *Treponema*, and the PEB enterotype dominated by *Prevotella* and *Mitsuokella*. Indeed, *Ruminococcus* did not differentiate PWE1 and PWE2, while it is a major marker between PEA and PEB. Interestingly, the PWE2 enterotype partially corresponds to the PEB enterotype with greater relative abundance of *Prevotella* and *Mitsuokella*, but the relative abundance in *Treponema* in PWE2 pointed towards PEA. These differences in the enterotypes at post-weaning could be related to differences in the diets. However, despite differences in the diet composition and the breeds during the finishing stage, the FE1 and FE2 enterotypes observed from 119 to 152 days of age in our study were consistent with previous observations [13,25].

Enterotypes are unstable, yet robust. Indeed, the pigs moved from an enterotype defined at 52 days of age to another one defined at 99 days of age but the pigs’ affiliation to an enterotype at 99 days was not determined by its affiliation to a specific enterotype at 52 days. Similar results were described between enterotypes in the post-weaning period [11] and between weaning and finishing [13]. Furthermore, during the finishing stage, pigs’ affiliation to an enterotype was not stable and they could switch enterotypes several times from 99 to 154 days. More precisely, this instability has already been observed on 1057 pigs between 23 and 26 weeks of age where the same enterotypes as in the current experiment have been identified [25]. Interestingly, this indicates that the enterotypes identified here are robust, as the later pigs originated from various farms. Similarly, pigs affiliated to an enterotype at 118 days of age were split into the two enterotypes defined at 196 days [13]. Such instability was already assumed by Knight et al. [26] in healthy humans.

The enterotypes also differ functionally, especially during the finishing stage. Indeed, the PWE1 and PWE2 enterotypes were functionally relatively similar to each other, with only four other pathways that were significantly different: ppGpp biosynthesis, superpathway of (Kdo)2-lipid A biosynthesis, glucose, glucose-1-phosphate degradation, and adenosylcobalamin salvage from cobinamide I (Table S4). In contrast the PICRUST algorithm predicts that seven MetaCyc pathways have a higher abundance in the FE1 enterotype (Figure S6): three of them are degrading pyruvate (methylerythritol phosphate pathway I, pyruvate fermentation to propanoate, and superpathway of butanediol biosynthesis) and three others produced Acetyl-CoA (reductive acetyl coenzyme A pathway, l-glutamate degradation V (via hydroxyglutarate), and myo-inositol degradation I). The last pathway was adenosylcobalamin salvage from cobinamide I which was already different between the post-weaning enterotypes (Table S4). Pyruvate also seems to be pivotal for the 40 pathways overrepresented in the FE2 enterotype, where it may be produced by neoglucogenesis, by the reductive TCA cycle I, by the degradation of hexitol or hexuronide, by the Enterner–Douroff pathway, or by the degradation of aspartate. Several carbohydrate degradation pathways were also overrepresented in FE2, namely fructose, hexitol, lactose and galactose, and rhamnose. The coenzyme A biosythesis I was overrepresented, and several fermentation pathways (homo- and hetero-lactic, mixed acid, and the Bifidobacterium shunt). Surprisingly, the polyamine biosynthesis I and II were also overrepresented in FE2, yet no adverse impact on growth was observed. In our experiment, we identified an intermediate enterotype (FE3) at 119 days that illustrated the switch between enterotypes with genus relative abundance in-between FE1 and FE2. Pigs in the intermediate enterotype had greater performance compared to the pigs in FE2 during the week before the feces collection. Similarly, Ramayo–Caldas et al. [12] revealed a growth performance difference between the two enterotypes identified in younger pigs (60 days of age). Therefore, we can assume that enterotype switches could be related to transient performance modifications. However, the difference of 850 g on 25 kg pigs previously observed remained quite low [12], and in our study, the performance of pigs in FE1 and FE2 enterotypes did not differ in accordance with previous findings [25]. This suggests that the relationship between enterotypes and an animal’s performance is not so clear.

In this study, the DON exposure impacted the ADFI, ADG, and FCR of the pigs at 119 and 140 days of age as previously detailed by Serviento et al. [27], but no impact on the performances was observed after the two-week recovery at 154 days of age. In addition, a transient impact on the microbiota composition has been observed [28]. However, in our experimental conditions, DON exposure did not impact the enterotype repartition among the animals nor their possible instability, suggesting that the enterotypes are insensitive to DON exposure. From this experiment, we could not identify any cause why pigs switched from an enterotype to another.

At 99 days of age, more females clustered in the FE2 enterotype compared to the FE1 enterotype, while the castrated males were equally distributed. This interaction between the enterotype and the sex type in the beginning of the finishing stage no longer existed at the end of the experiment. It seems that the age interacts with the sex type in terms of microbiota composition. Accordingly, the microbial difference observed between male and female pigs disappeared after 63 days of age [23].

## 5. Conclusions

In conclusion, pig gut microbiota evolved mainly from 52 to 99 days of age with an increased abundance of Streptococcaceae and a decreased abundance of Lactobacillaceae. At 52 days, two enterotypes dominated either by *Lactobacillus* or by *Prevotella*–*Sarcina* were identified. They differed from the enterotypes determined from 99 to 154 days and dominated either by *Lactobacillus* or by *Turicibacter*–*Clostridium sensu stricto*. The enterotype definition was time-dependent. Previous findings about the relationships between enterotypes and improved performance in pigs might have suggested that adding bacterial supplements in the feed could possibly help in modifying the enterotypes and the performance. However, our study demonstrated that the enterotypes defined between 99 and 154 days of age were independent of the growth and feeding performance. Thus, we would advise the careful use of enterotypes when trying to relate them to performance differences in an industrial context.

## Figures and Tables

**Table 1:** Age effect on female pigs’ fecal microbiota diversity and phyla relative abundance.

	Days of Age				
Item	52	99	119	140	154
Number of samples ^1^	32	64	60	61	65
					
Diversity indexes					
Nb of OTU	2 709 ± 323 ^a^	3 666 ± 363 ^b^	3 600 ± 334 ^b^	3 719 ± 288 ^b^	3 726 ± 298 ^b^
Shannon index	6.67 ± 0.17 ^a^	7.14 ± 0.16 ^b^	7.15 ± 0.13 ^b^	7.19 ± 0.11 ^b^	7.17 ± 0.11 ^b^
Chao1	3 707 ± 506 ^a^	5 229 ± 876 ^bc^	4 966 ± 731 ^c^	5 119 ± 583 ^bc^	5 276 ± 670 ^b^
ACE	3 866 ± 563 ^a^	5 559 ± 980 ^b^	5 227 ± 814 ^c^	5 403 ± 649 ^bc^	5 602 ± 772 ^b^
Simpson	0.994 ± 0.003 ^a^	0.997 ± 0.001 ^b^	0.996 ± 0.001 ^c^	0.997 ± 0.001 ^bc^	0.997 ± 0.001 ^b^
InvSimpson	209 ± 64 ^a^	310 ± 68 ^b^	284 ± 78 ^c^	309 ± 75 ^bc^	324 ± 86 ^b^
Fisher	949 ± 165 ^a^	1 512 ± 242 ^b^	1 467 ± 217 ^b^	1 543 ± 191 ^b^	1 548 ± 200 ^b^
					
Phyla relative abundance (%)					
Firmicutes	72.09 ± 7.61 ^a^	80.31 ± 5.52 ^b^	78.15 ± 5.05 ^b^	78.44 ± 5.89 ^b^	78.30 ± 6.77 ^b^
Bacteroidetes	22.56 ± 7.82 ^a^	15.02 ± 5.26 ^bc^	16.18 ± 5.14 ^b^	15.17 ± 5.66 ^bc^	13.26 ± 3.90 ^c^
Spirochaetes	0.34 ± 0.88 ^a^	0.18 ± 0.28 ^a^	0.47 ± 0.45 ^b^	0.70 ± 0.49 ^c^	1.33 ± 1.28 ^d^
Actinobacteria	0.66 ± 1.12 ^a^	0.71 ± 1.16 ^a^	0.26 ± 0.20 ^b^	0.20 ± 0.09 ^b^	0.21 ± 0.15 ^b^
Proteobacteria	0.58 ± 0.51	0.48 ± 0.34	0.44 ± 0.22	0.39 ± 0.24	0.53 ± 0.33
Fibrobacteres	<0.1	<0.1	<0.1	<0.1	<0.1
Tenericutes	<0.1	<0.1	<0.1	<0.1	<0.1
Unclassified	3.75 ± 2.15 ^a^	3.28 ± 1.57 ^a^	4.47 ± 1.89 ^b^	5.06 ± 1.84 ^b^	6.29 ± 2.88 ^c^
					
Firmicutes/Bacteroidetes	3.84 ± 2.10 ^a^	6.27 ± 3.05 ^bc^	5.52 ± 2.43 ^c^	5.98 ± 2.45 b ^c^	6.66 ± 2.86 ^b^

^a–d^ Least square means ± standard deviation within a row with different superscript significantly differ (*p* < 0.05) in Kruskal–Wallis tests. ^1^ At 99, 119, 140, and 154 d, the samples were collected on pigs raised in two consecutive batches. At 52 d, pigs’ fecal samples from the second replicate only were collected.

**Table 2:** Effect of the finishing enterotypes on the performance of the pigs.

Age at Fecal Sampling (day) ^1^		Enterotype								
		FE1		FE2		FE3				
	Trait _Days of Age_	mean	SE	mean	SE	mean	SE	RSD ^2^	Tested Factors	Statistics ^3^
99	No. of pigs	86		97						
	ADFI_99_–_113_ (kg/day)	2.45	0.04	2.42	0.04	-	-	0.30	E, R, I, S	I
	ADG_99_–_113_ (kg/day)	0.86	0.02	0.87	0.02	-	-	0.12	E, R, I, S	I
	FCR_99_–_113_	2.89	0.06	2.84	0.05	-	-	0.44	E, R, I, S	I
										
119	No. of pigs	7		33		26				
	ADFI_113_–_119_ (kg/day)	2.08	0.12	1.97	0.06	2.12	0.07	0.31	E, R, I, D	R, I, D
	ADG_113_–_119_ (kg/day)	0.84 ^ab^	0.08	0.65 ^b^	0.04	0.89 ^a^	0.04	0.21	E, R, I, D	E, D
	FCR_113_–_119_	2.96	1.25	4.21	0.65	3.04	0.68	3.18	E, R, I, D	D
										
	ADFI_119_–_134_ (kg/day)	2.50	0.11	2.59	0.06	2.58	0.06	0.27	E, R, I, D	R, I
	ADG_119_–_134_ (kg/day)	1.01	0.06	1.04	0.03	1.04	0.04	0.16	E, R, I, D	I
	FCR_119_–_134_	2.51	0.46	2.43	0.24	2.89	0.25	1.18	E, R, I, D	-
										
140	No. of pigs	28		44						
	ADFI_134_–_140_ (kg/day)	2.61	0.07	2.55	0.06	-	-	0.37	E, R, I, D	I, D
	ADG_134_–_140_ (kg/day)	0.91	0.04	0.91	0.04	-	-	0.22	E, R, I, D	I, D
	FCR_134_–_140_	3.21	2.23	5.36	1.86	-	-	11.14	E, R, I, D	-
										
	ADFI_140_–_154_ (kg/day)	3.18	0.07	3.07	0.06	-	-	0.35	E, R, I, D	I
	ADG_140_–_154_ (kg/day)	1.12	0.04	1.10	0.03	-	-	0.02	E, R, I, D	D
	FCR_140_–_154_	2.89	0.08	2.86	0.07	-	-	0.42	E, R, I, D	-
										
154	No. of pigs	72		81						
	ADFI_140_–_154_ (kg/day)	3.34	0.05	3.35	0.05	-	-	0.38	E, R, I, S	I, S
	ADG_140_–_154_ (kg/day)	1.12	0.03	1.10	0.02	-	-	0.21	E, R, I, S	R
	FCR_140_–_154_	3.06	0.07	3.11	0.06	-	-	0.48	E, R, I, S	R, I, S

^a,b^ Least square means within a row with different superscript significantly differ (*p* < 0.05). ^1^ Feces were collected from females and castrated males at 99 and 154 days of age, and from females only at 119 and 140 days of age. Half of the female pigs received a DON-contaminated diet between 113 and 119, and between 134 and 140 days of age. ^2^ Residual standard deviation from an ANOVA model accounting for the enterotype (E), the replicate (R), the initial body weight of the pig at 99 days of age (I), the sex (S), and the DON challenge (D). All the interactions have been tested and none are significant. ^3^ Significant effects (*p* < 0.05).

## Supplementary Materials

The following are available online at https://www.mdpi.com/2076-2607/7/12/622/s1, Figure S1: Experimental design; Figure S2: Distribution of samples across sex, time points, replicates, and type of analysis; Figure S3: Non Metric Distance Scaling (NMDS) representation of the microbiota evolution across ages using Bray-Curtis distance in female pigs; Figure S4: Silhouette indexes, Calinski–Harabasz (CH) indexes and plots of the clusters of samples based on the genus relative abundance at 52, 99, 119, 140, and 154 days of age; Figure S5: Ratio of FE1 enterotypes at Day 154 in the CC, DC, CD, and DD experimental groups corresponding to pigs fed with a control finisher diet, exposed to a DON-contaminated diet between 113 d and 119 d, between 134 d and 140 d, and between both 113 d and 119 d and 134 d and 140 d of age, respectively; Figure S6: Significant MetaCyc pathway predicted by PICRUST; Table S1: Composition of experimental growing and finishing diets; Table S2: DON challenge effect on female pigs’ fecal microbiota diversity and phyla relative abundance; Table S3: *p*-values from Kruskal–Wallis tests for differences in genus relative abundance between the enterotypes at post-weaning (PWE1 vs. PWE2) and at finishing (FE1 vs. FE2); Table S4: Group file; Table S5: PICRUST prediction of Kegg orthologs; Table S6: PICRUST prediction of MetaCyc pathway abundance; Table S7: Statistics on the predicted MetaCyc abundance predicted by PICRUST.
