# Supplementary material for: Longitudinal Analysis of the Microbiota Composition and Enterotypes of Pigs from Post-Weaning to Finishing
Source: Microorganisms. 2019 Nov 28;7(12):622. doi: 10.3390/microorganisms7120622 (PMC6956163; doi:10.3390/microorganisms7120622)

Supplementary Figure S3

Methodology for the enterotype definition

To identify samples with similar bacterial composition, enterotypes at 52, 99, 119, 140 and 154 days were constituted following the methodology from Arumugam et al. (2011). Briefly, from the relative genus abundance, a Jensen-Shannon divergence matrix was calculated. The partitioning around medoïds clustering algorithm was applied and the optimal number of clusters (corresponding to enterotypes) was assessed using the Calinski-Harabasz index. To evaluate the relevance of the clustering on the real dataset, we randomized the dataset 100 times and compared the Silhouette indexes. A higher Silhouette index in the real dataset compared to the randomized ones confirmed a great clustering in the real dataset.

Supplementary Figure S3a

52 days of age

Dataset randomized

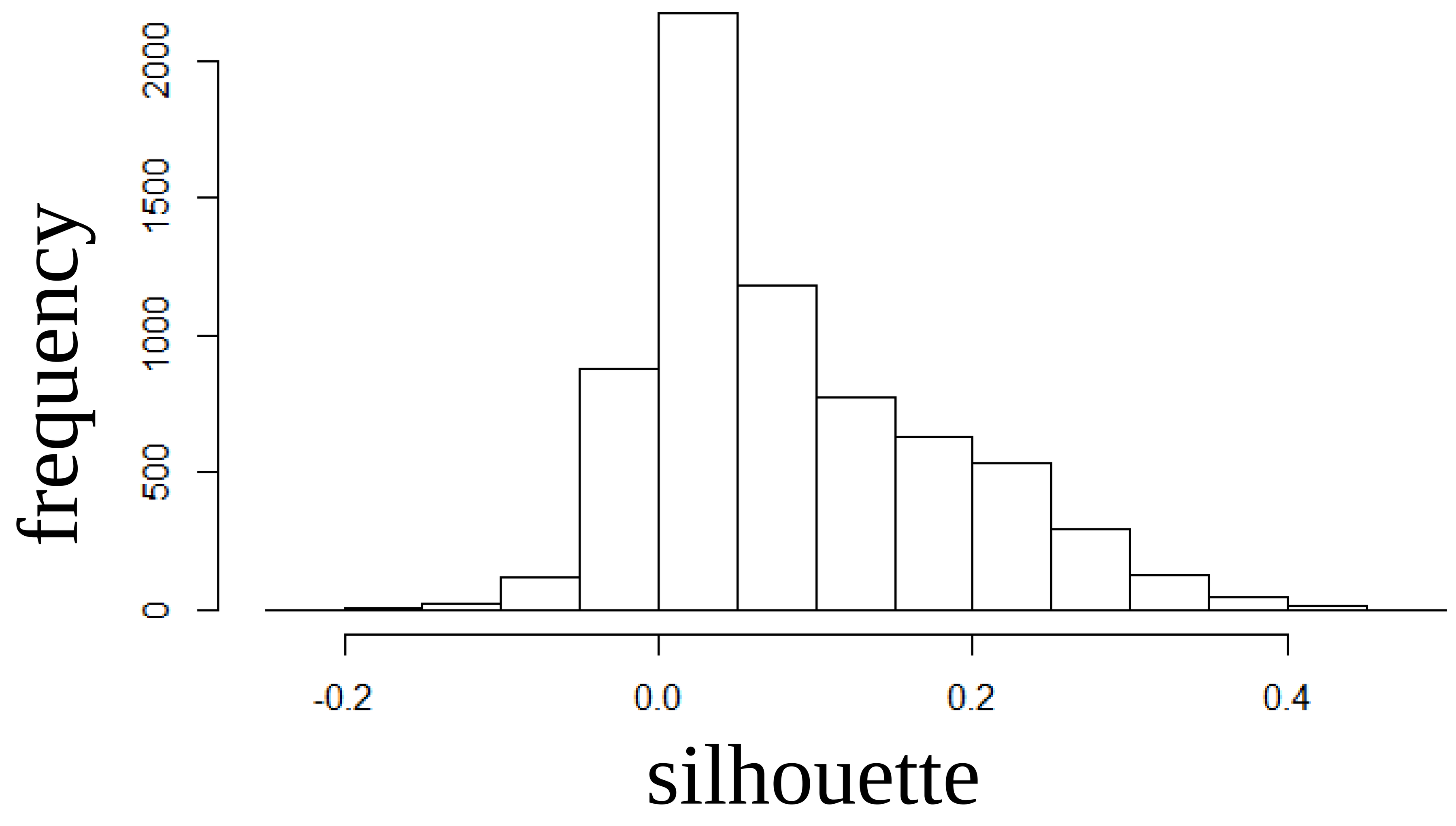

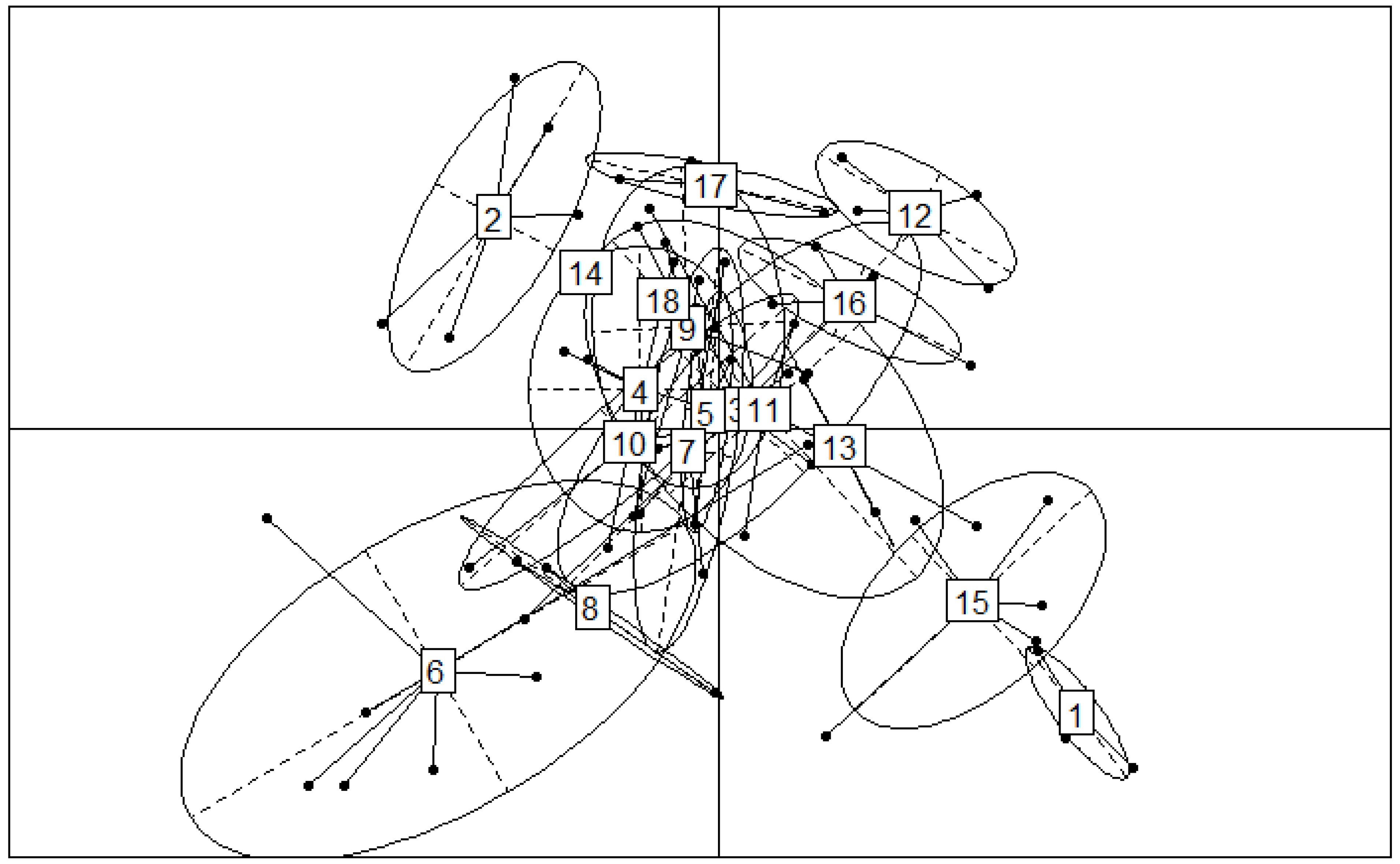

Real dataset

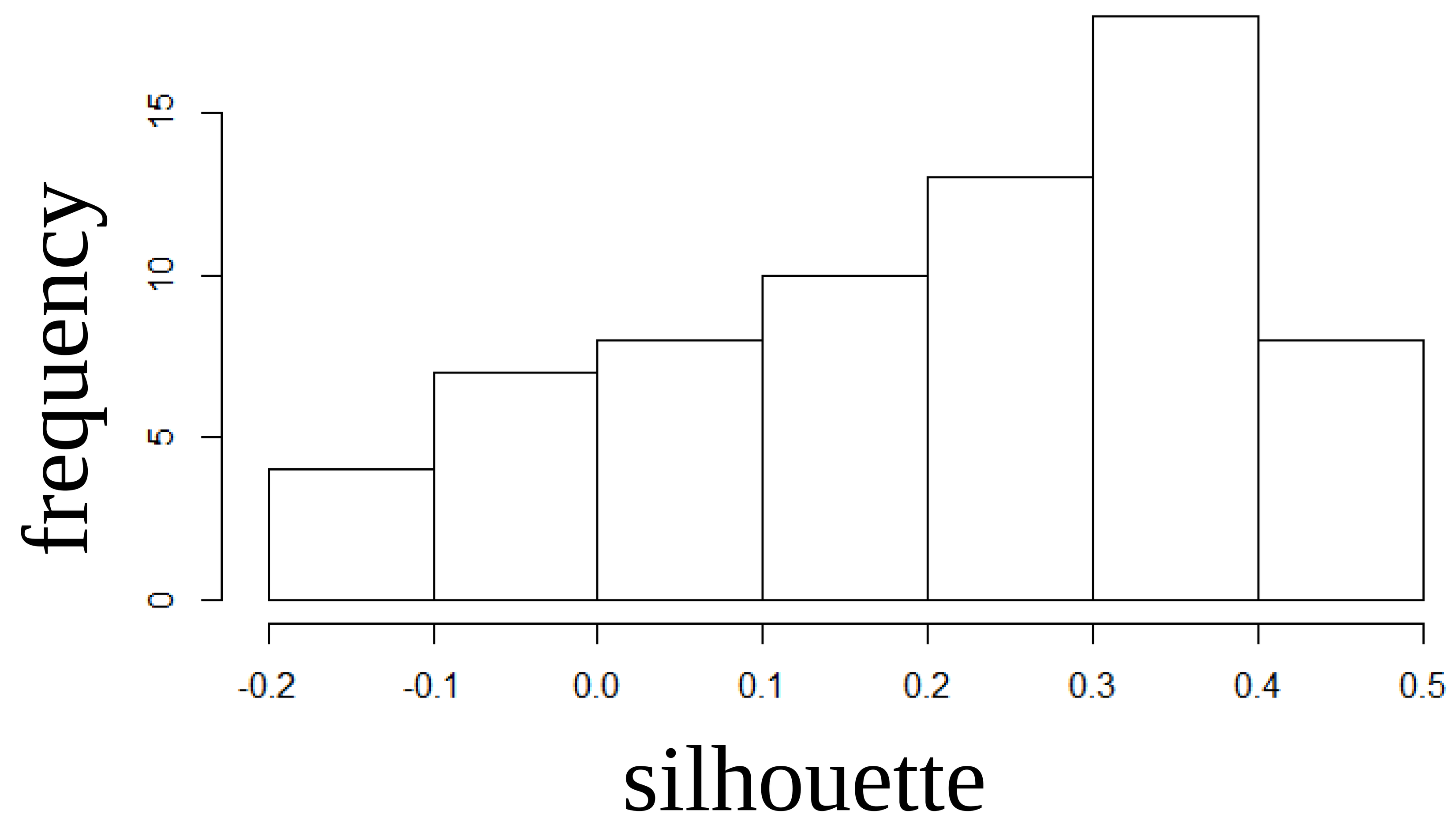

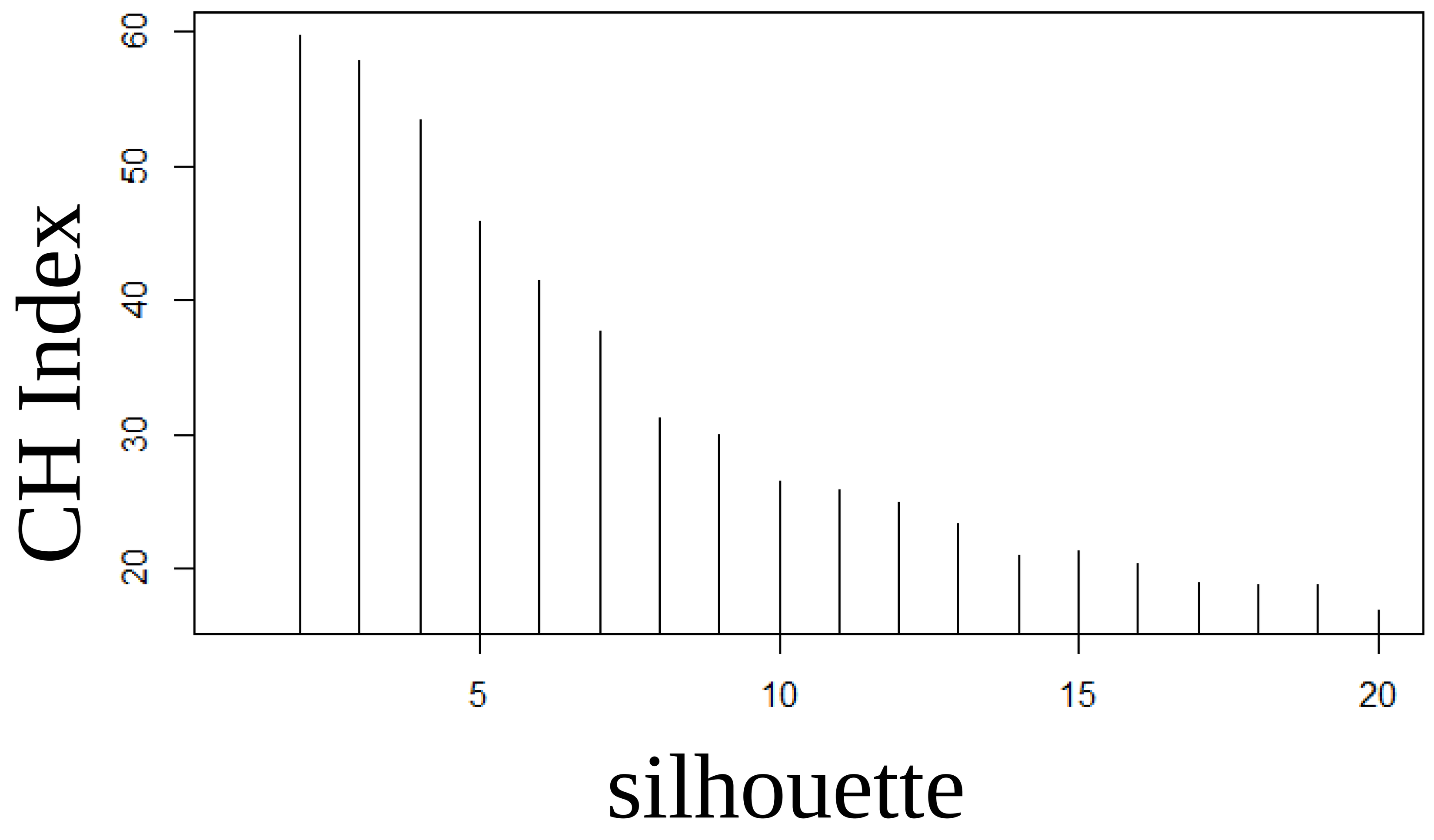

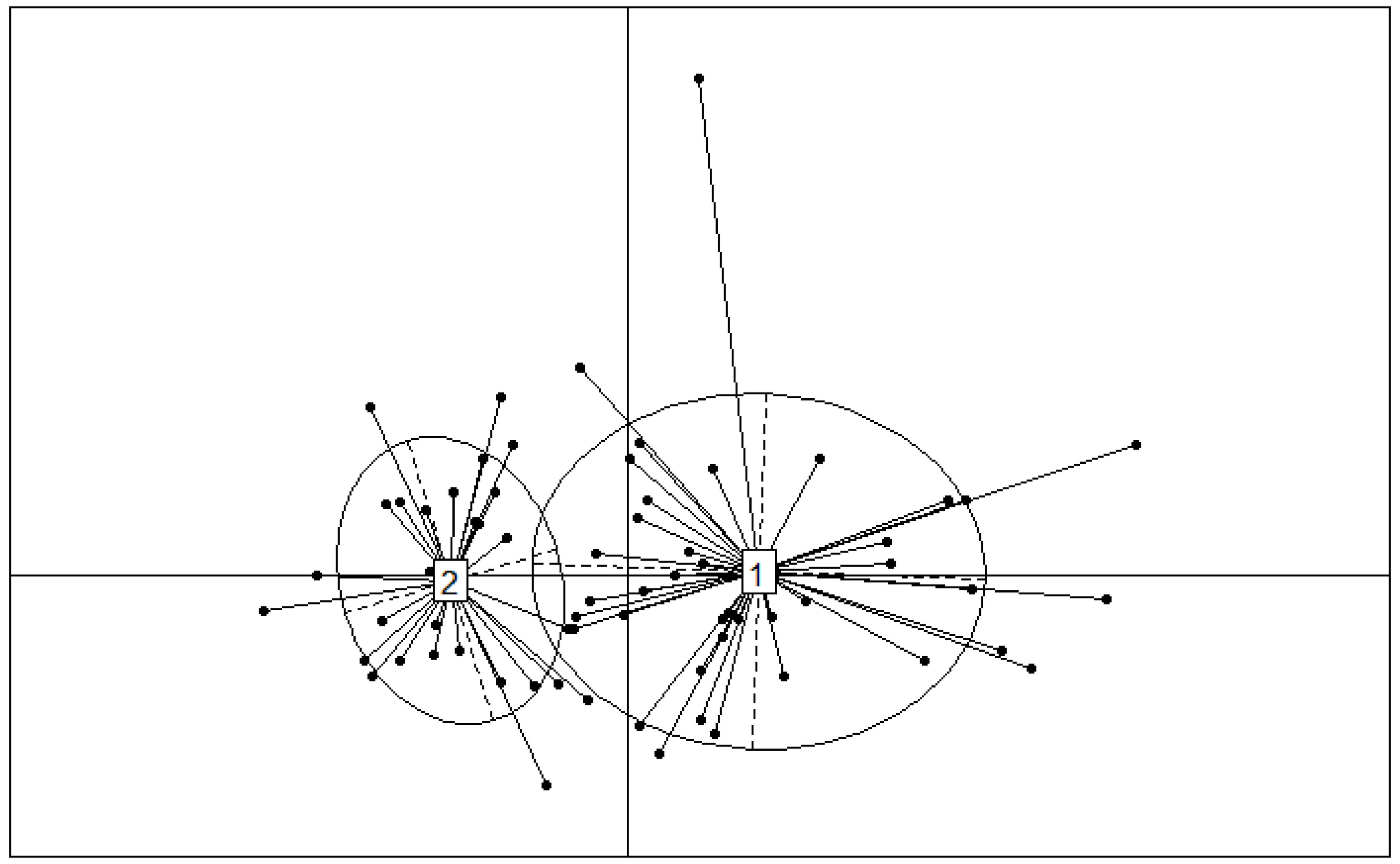

Supplementary Figure S3b

99 days of age

Dataset randomized

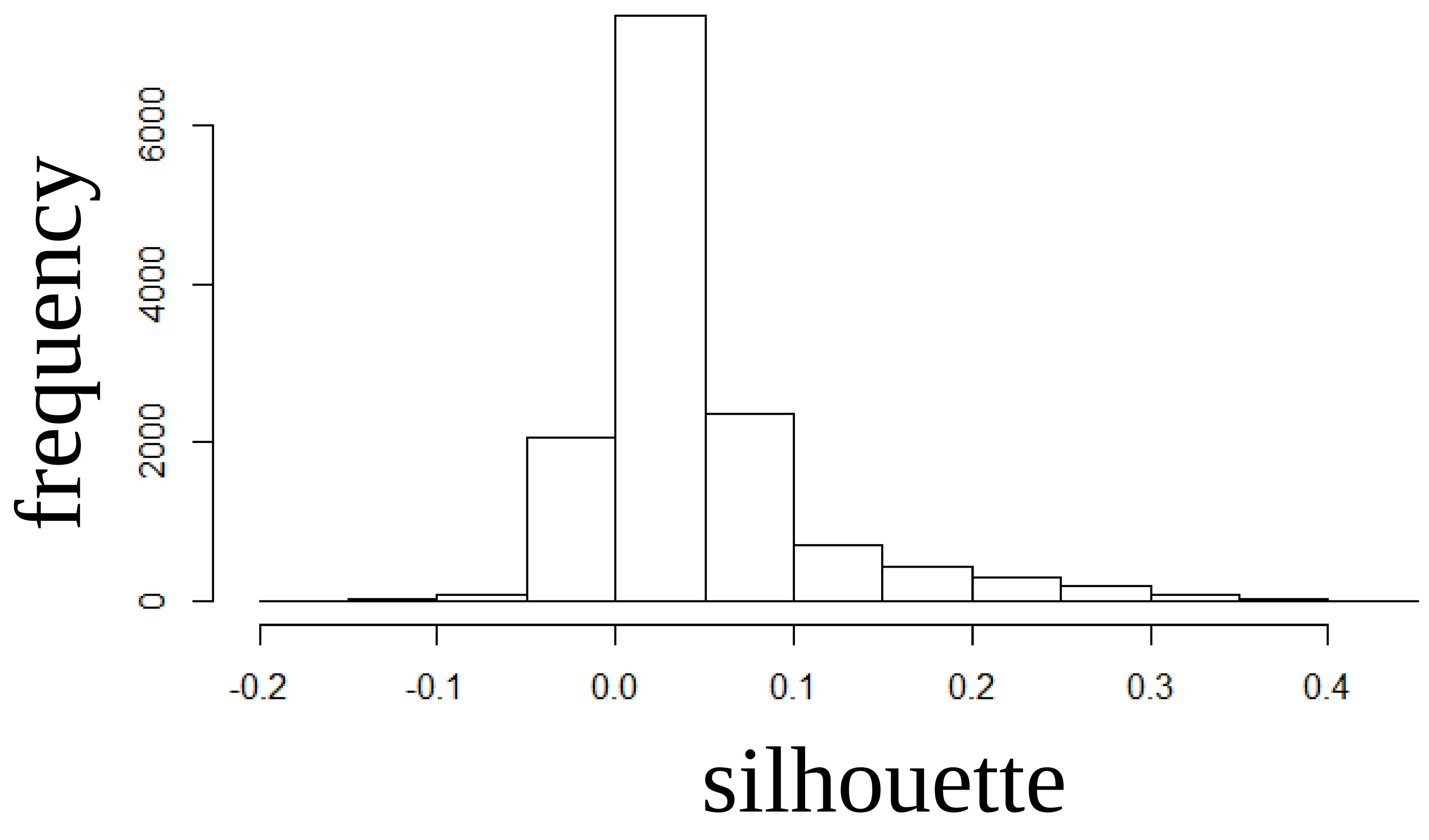

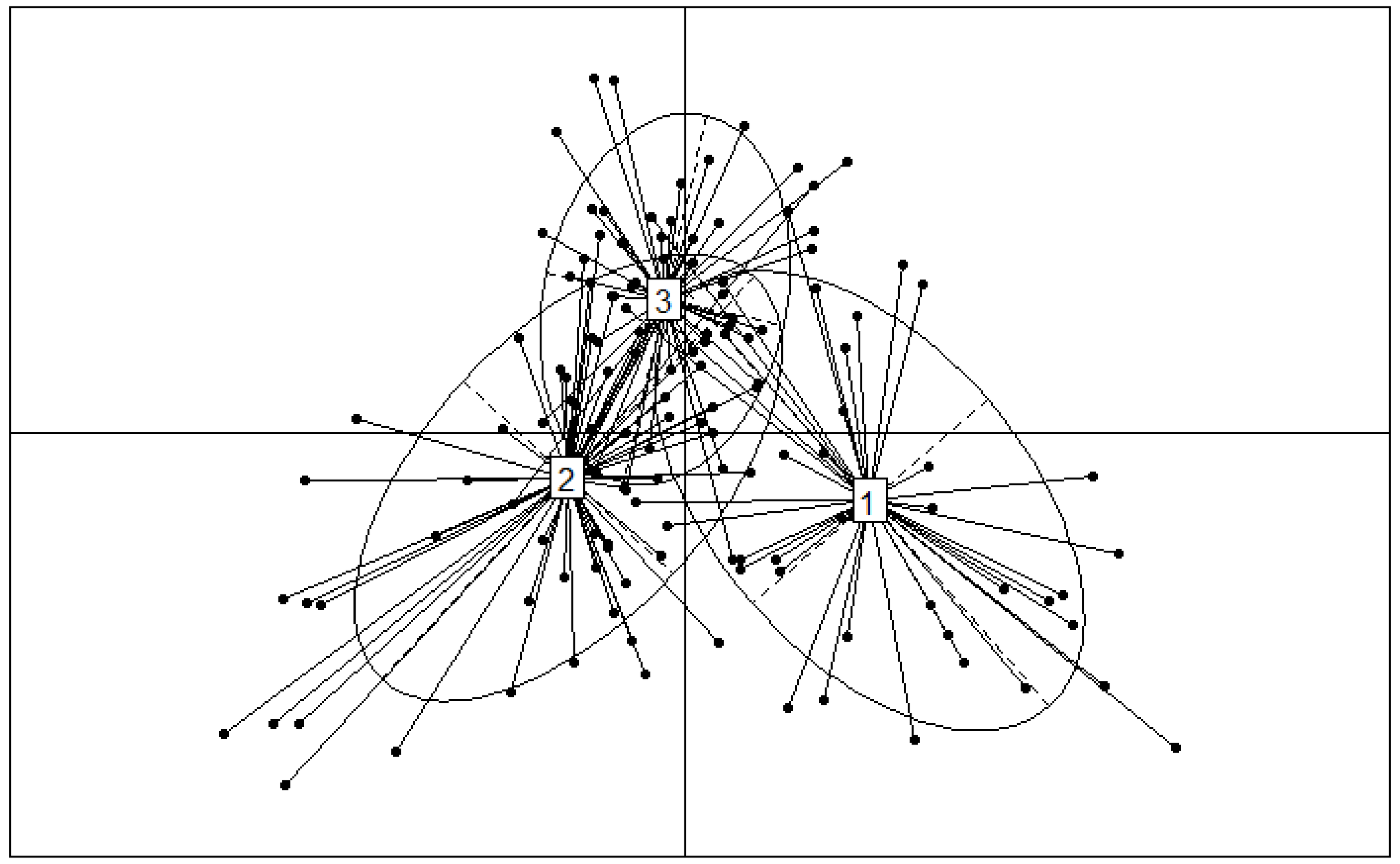

Real dataset

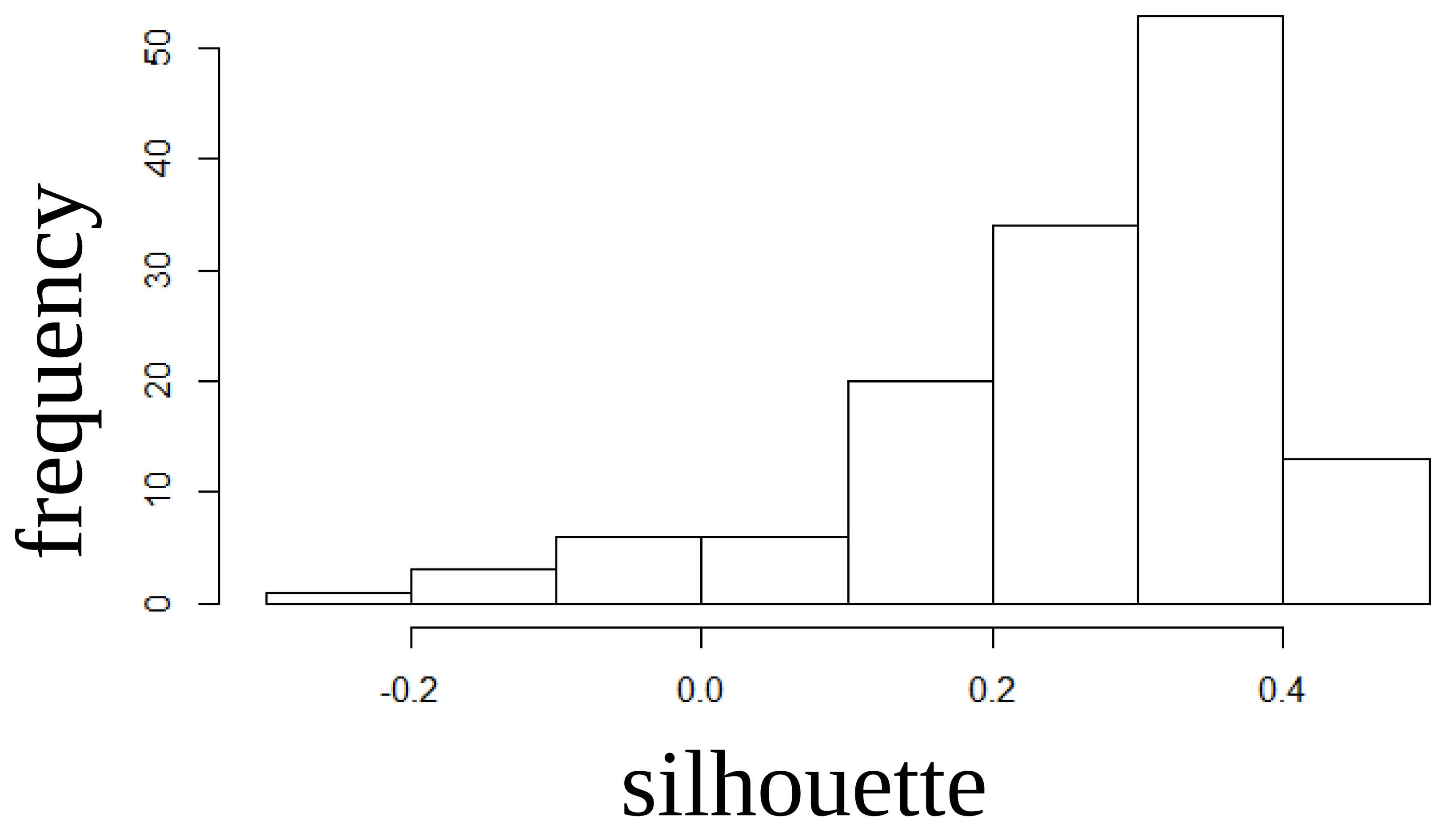

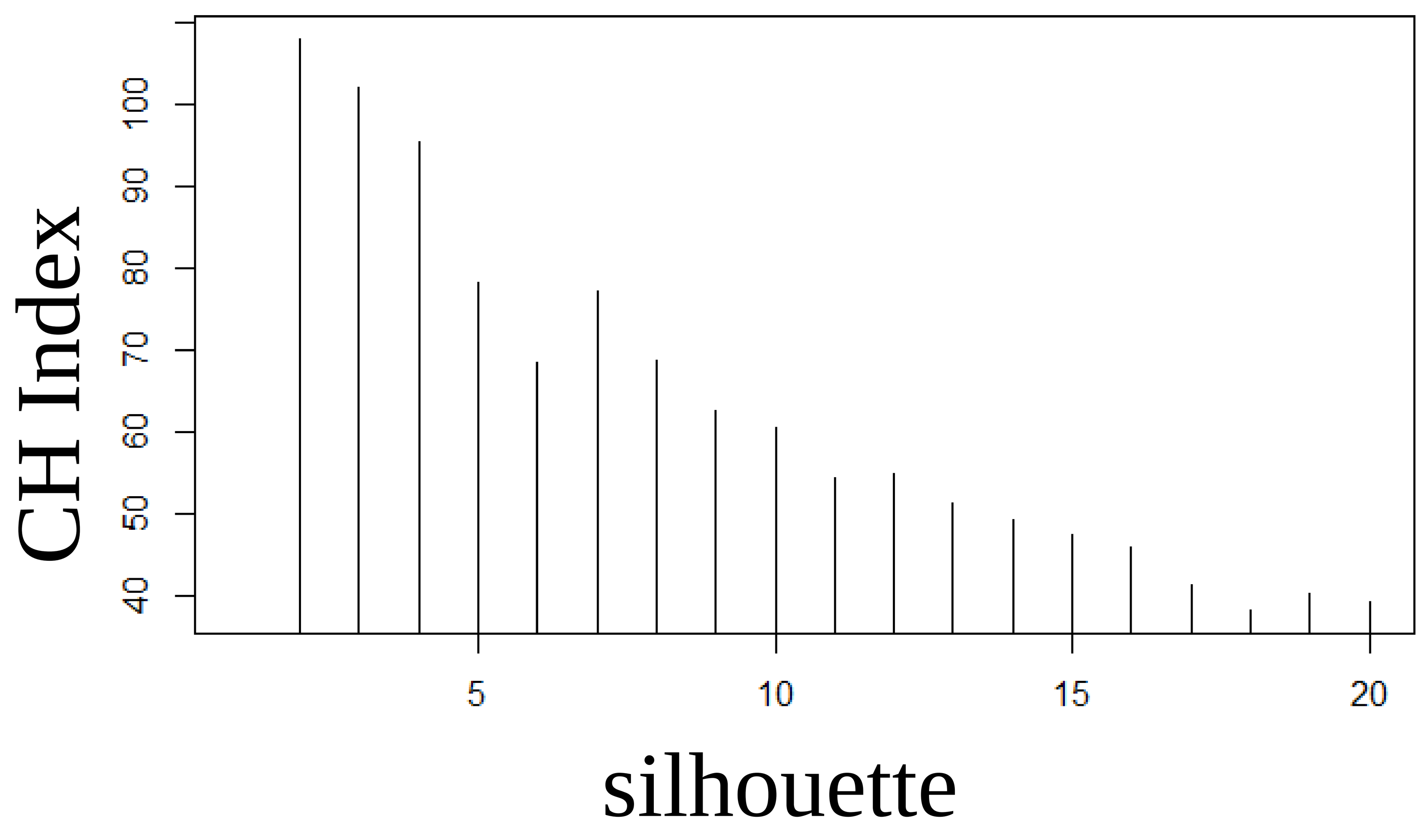

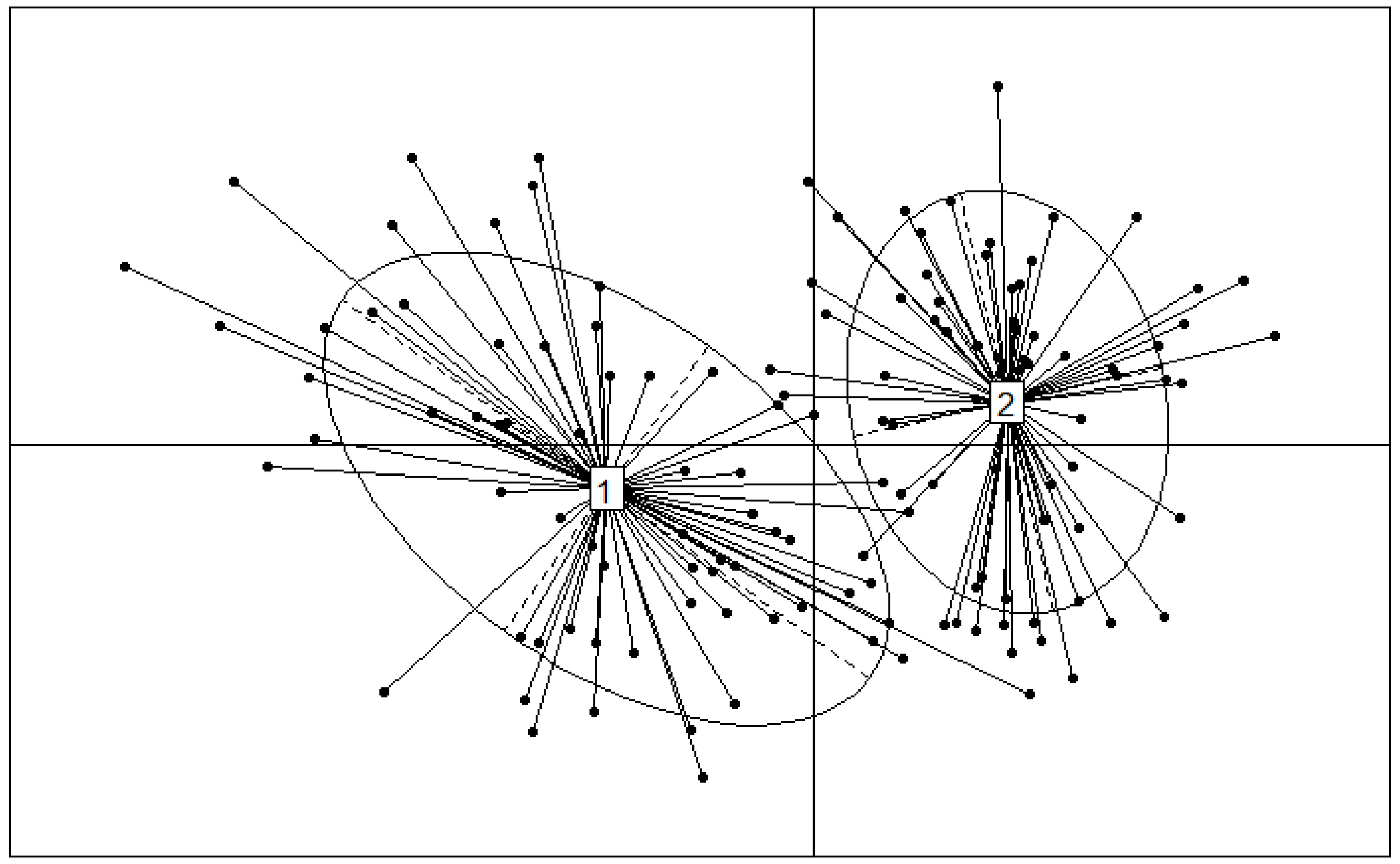

Supplementary Figure S3c

119 days of age

Dataset randomized

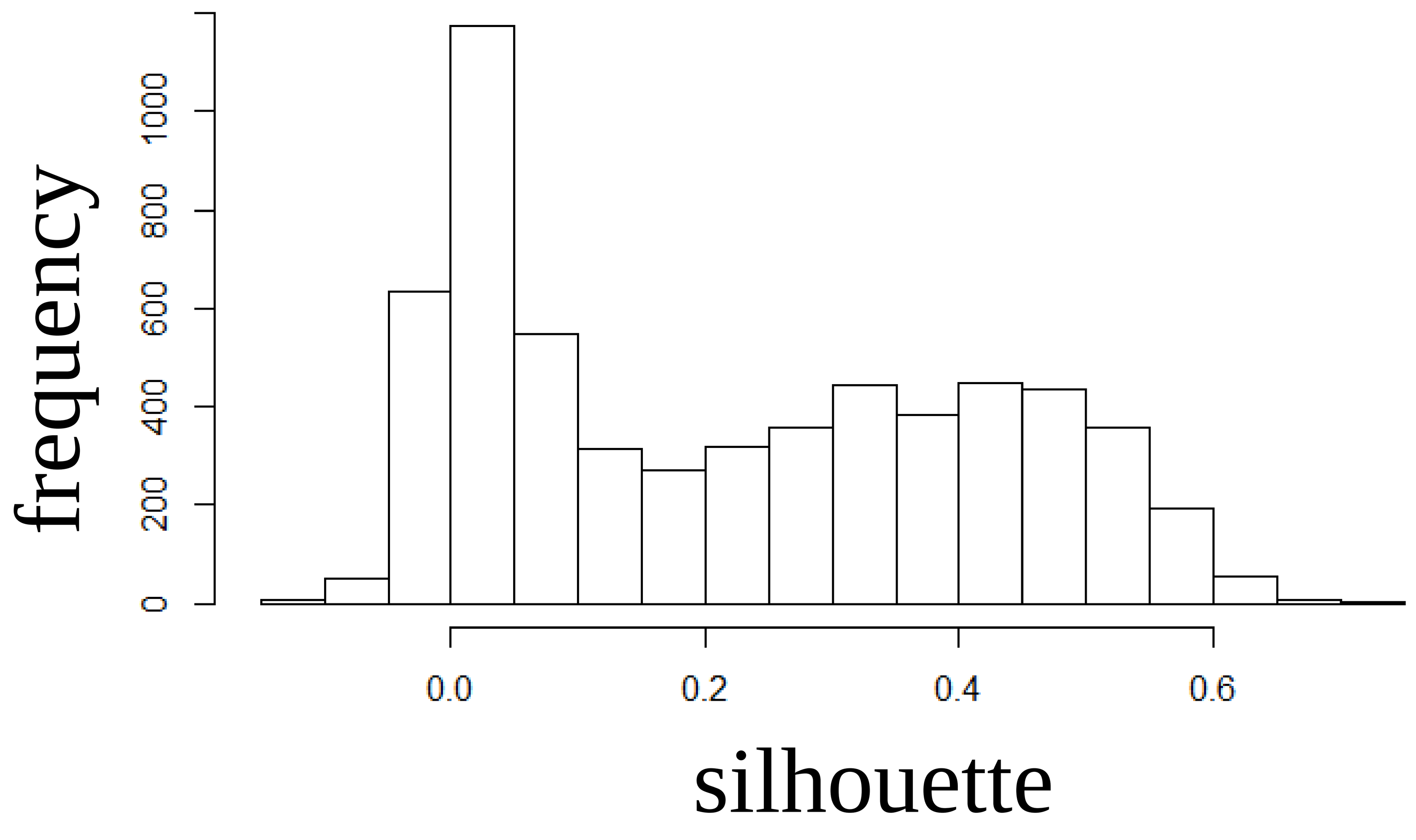

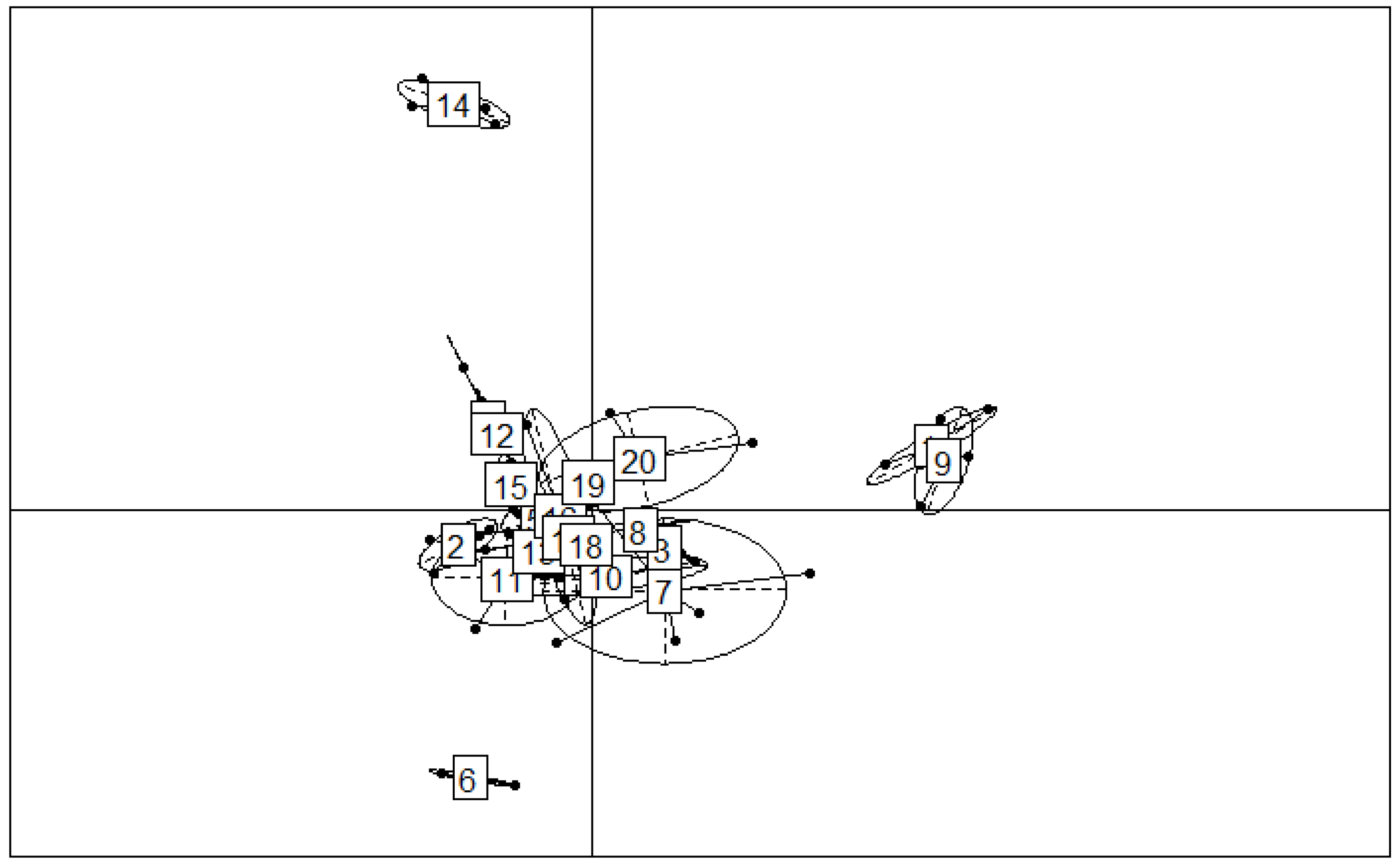

Real dataset

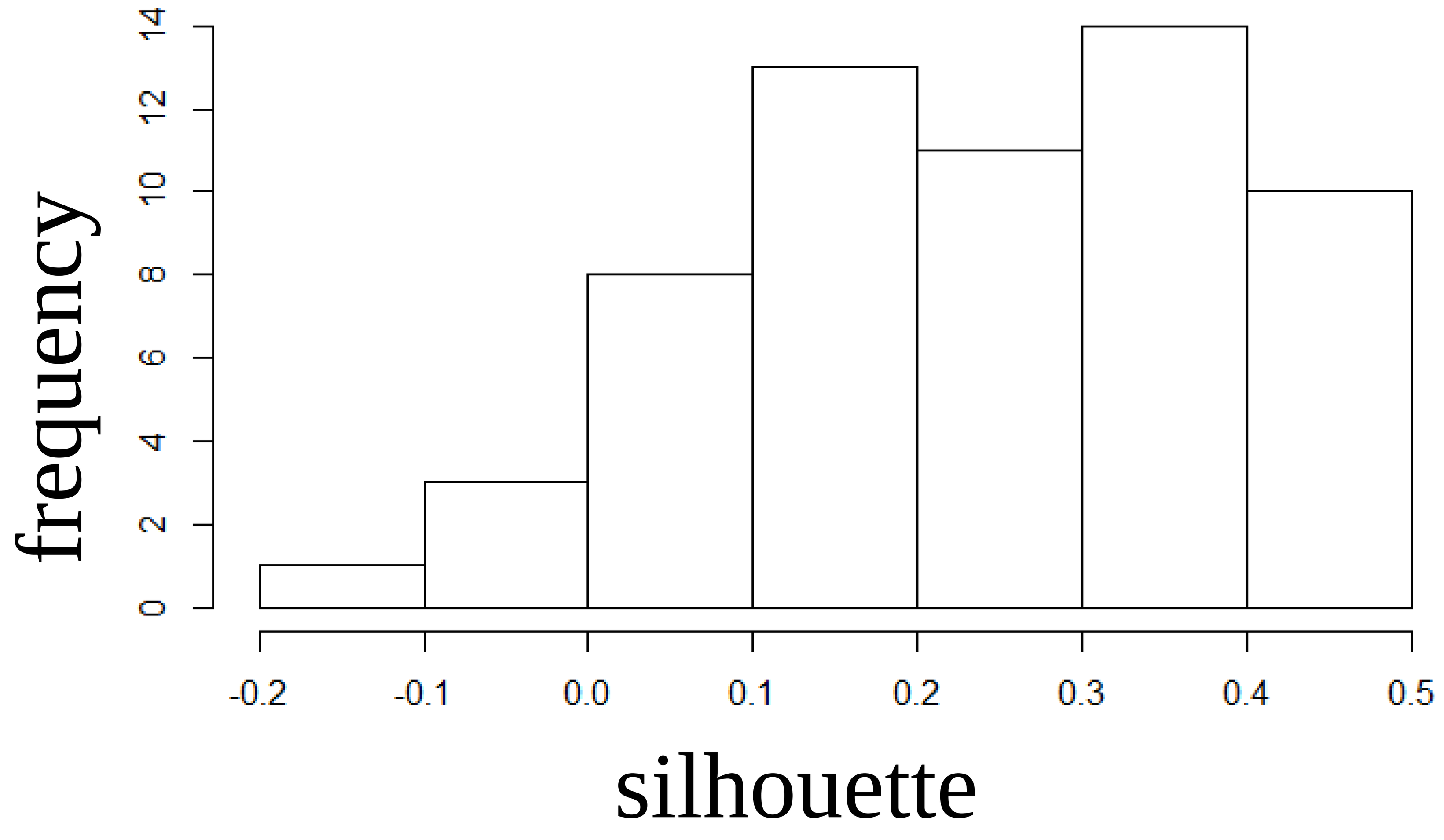

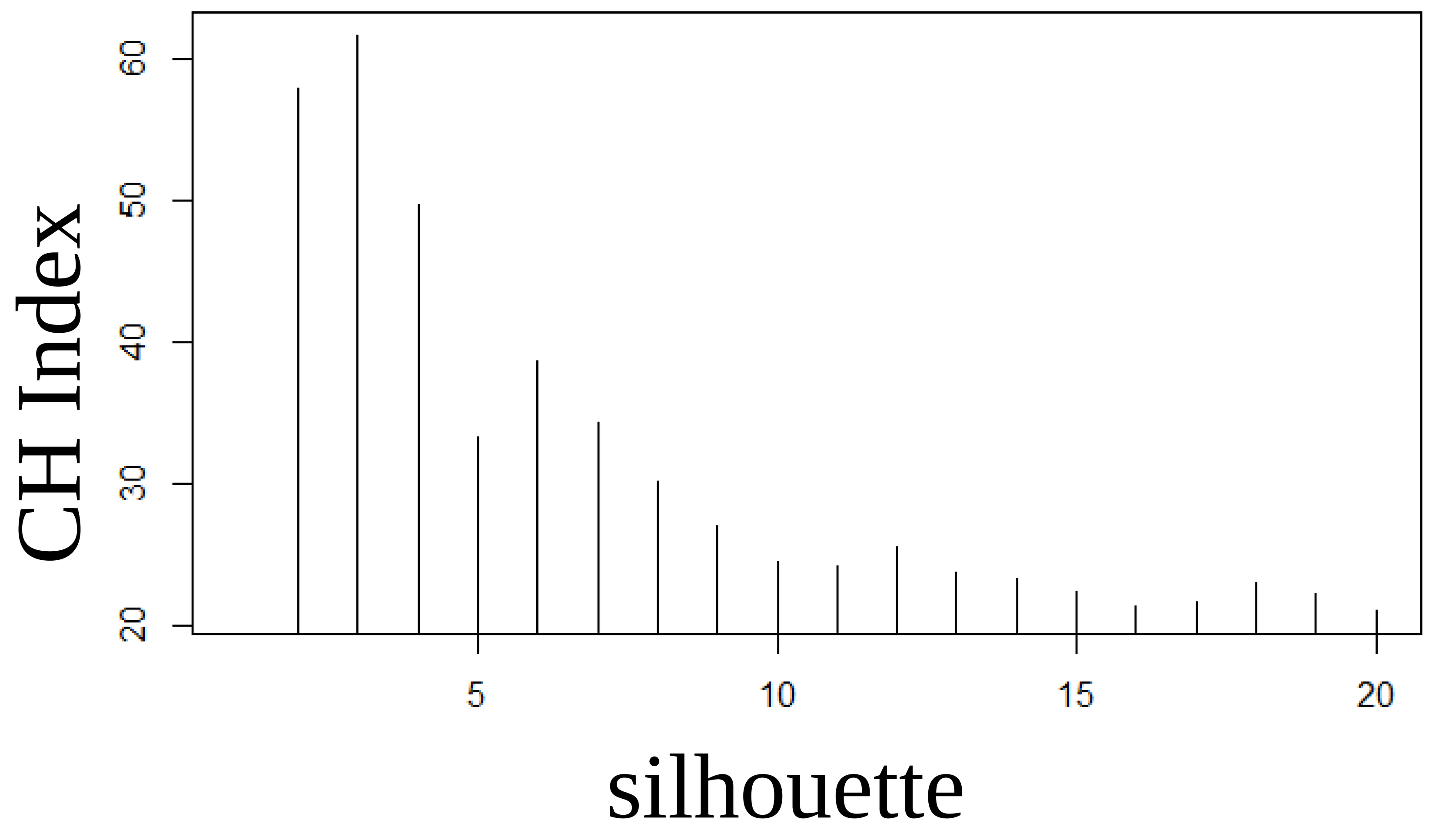

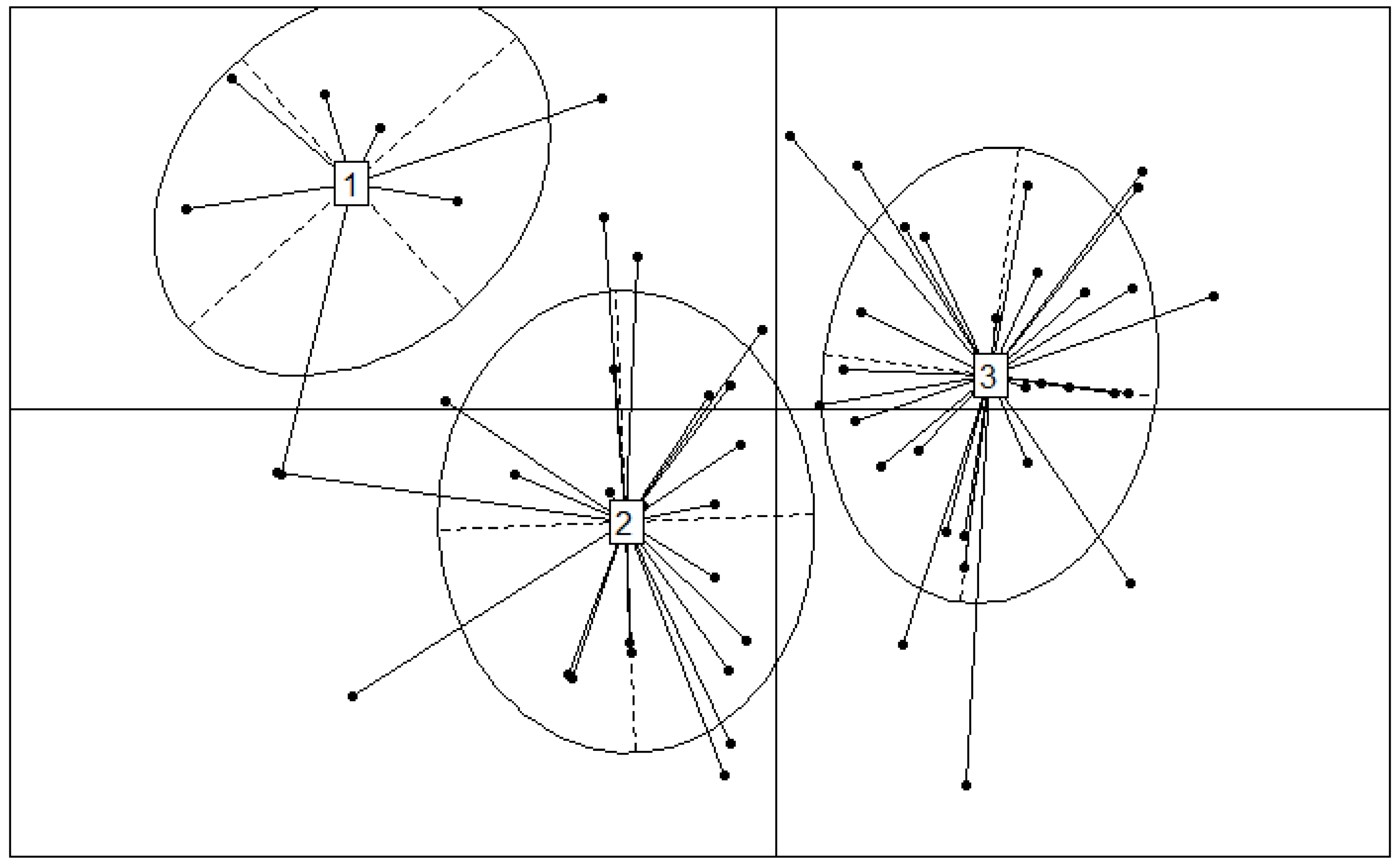

Supplementary Figure S3d

140 days of age

Dataset randomized

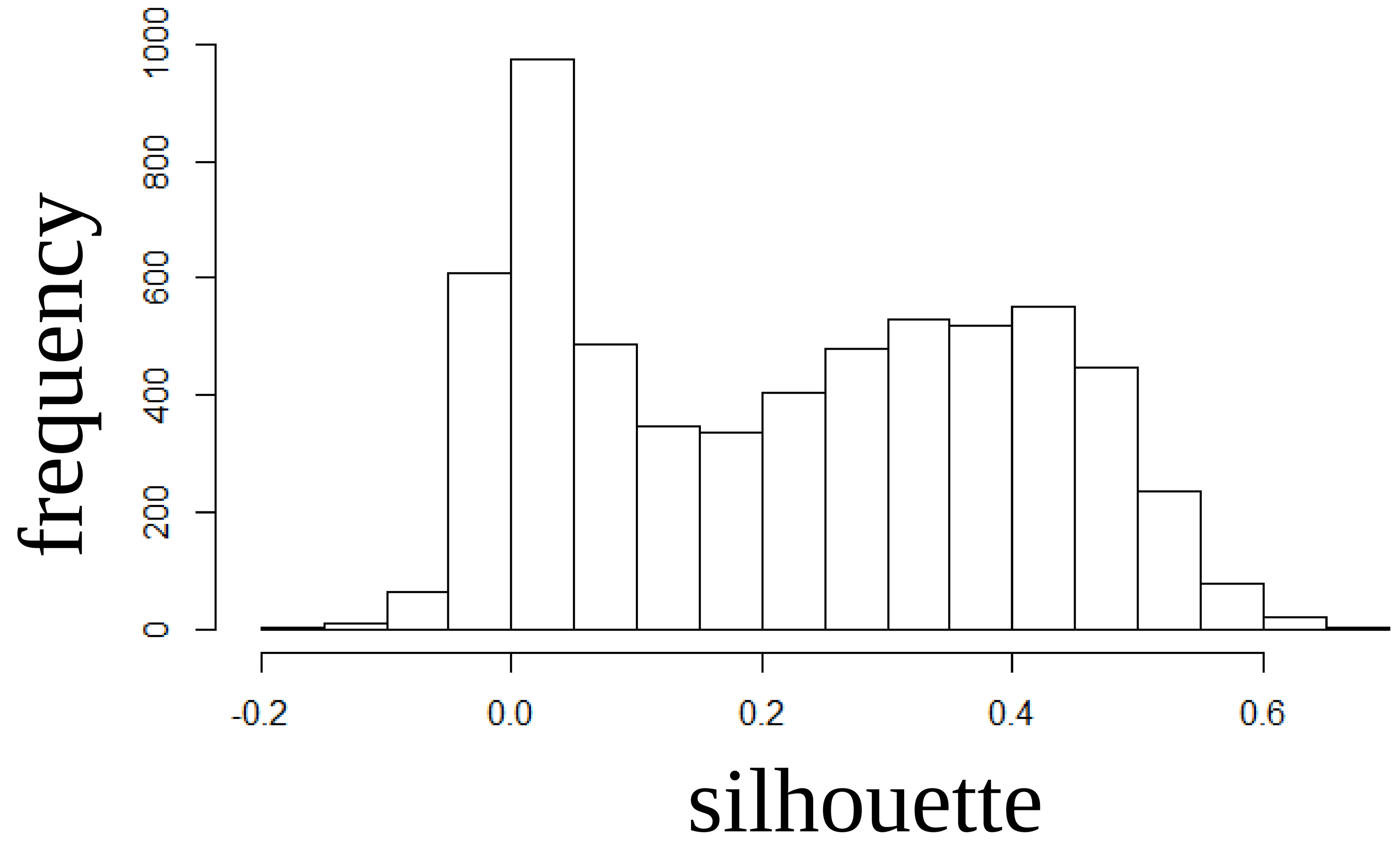

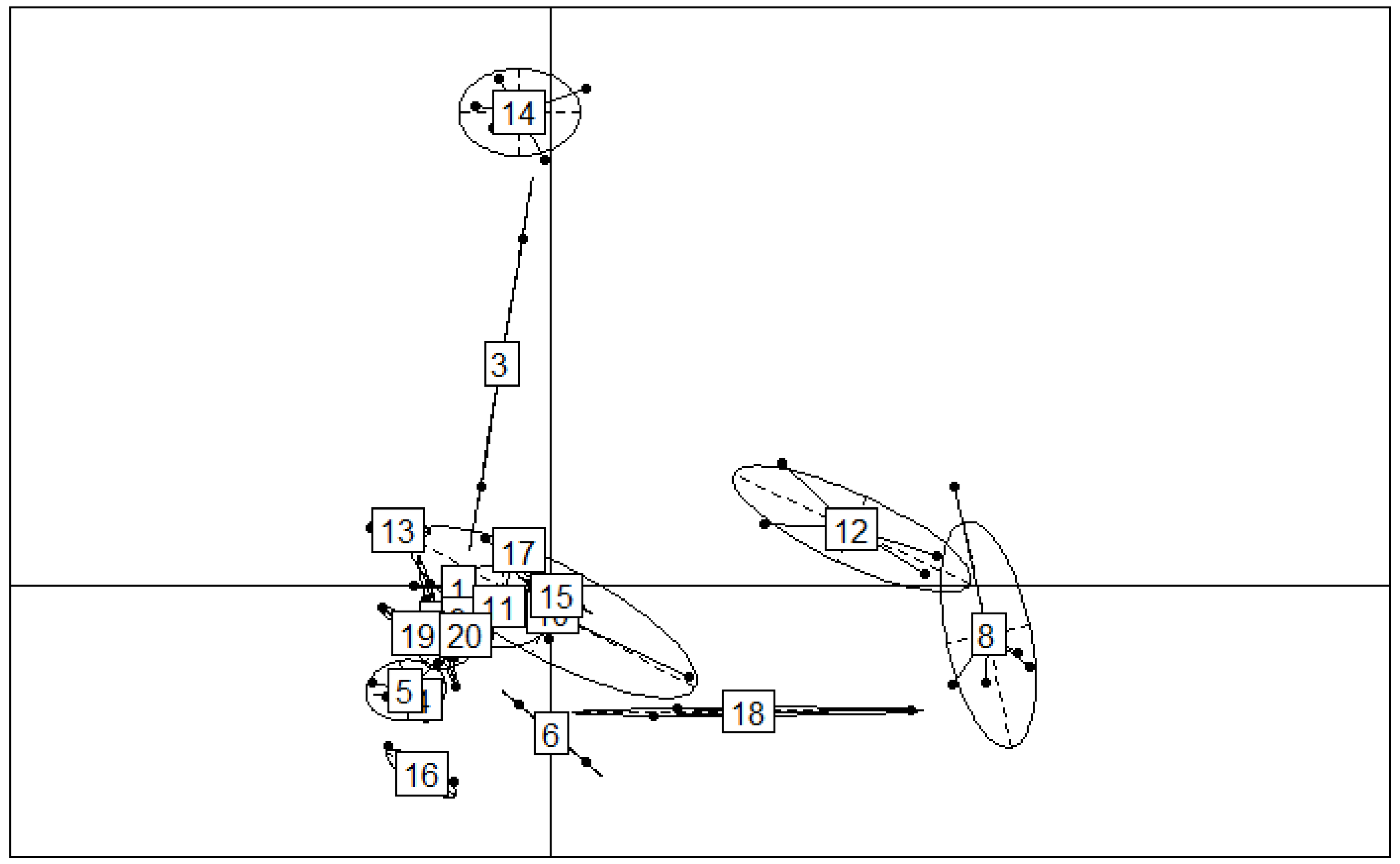

Real dataset

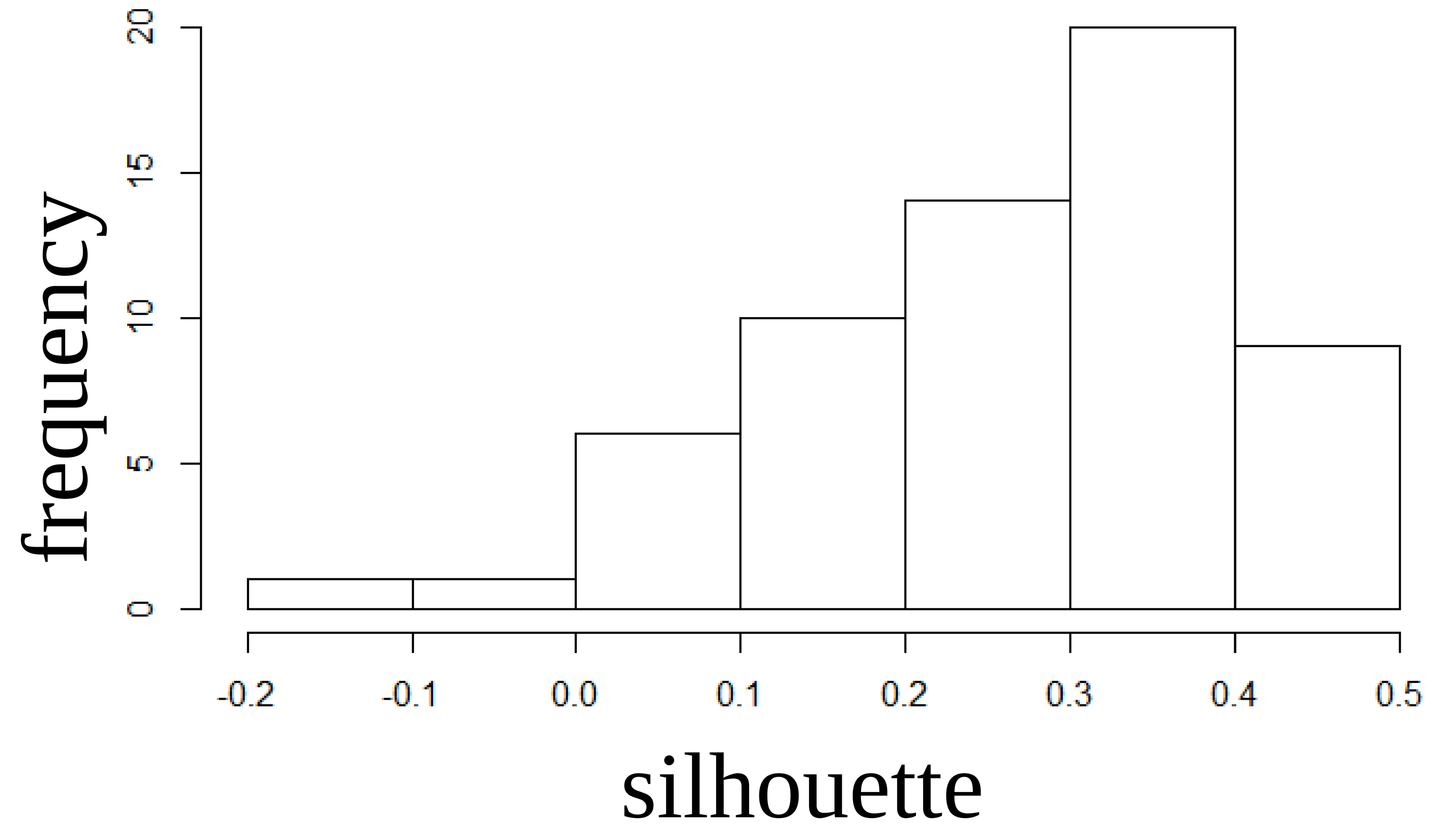

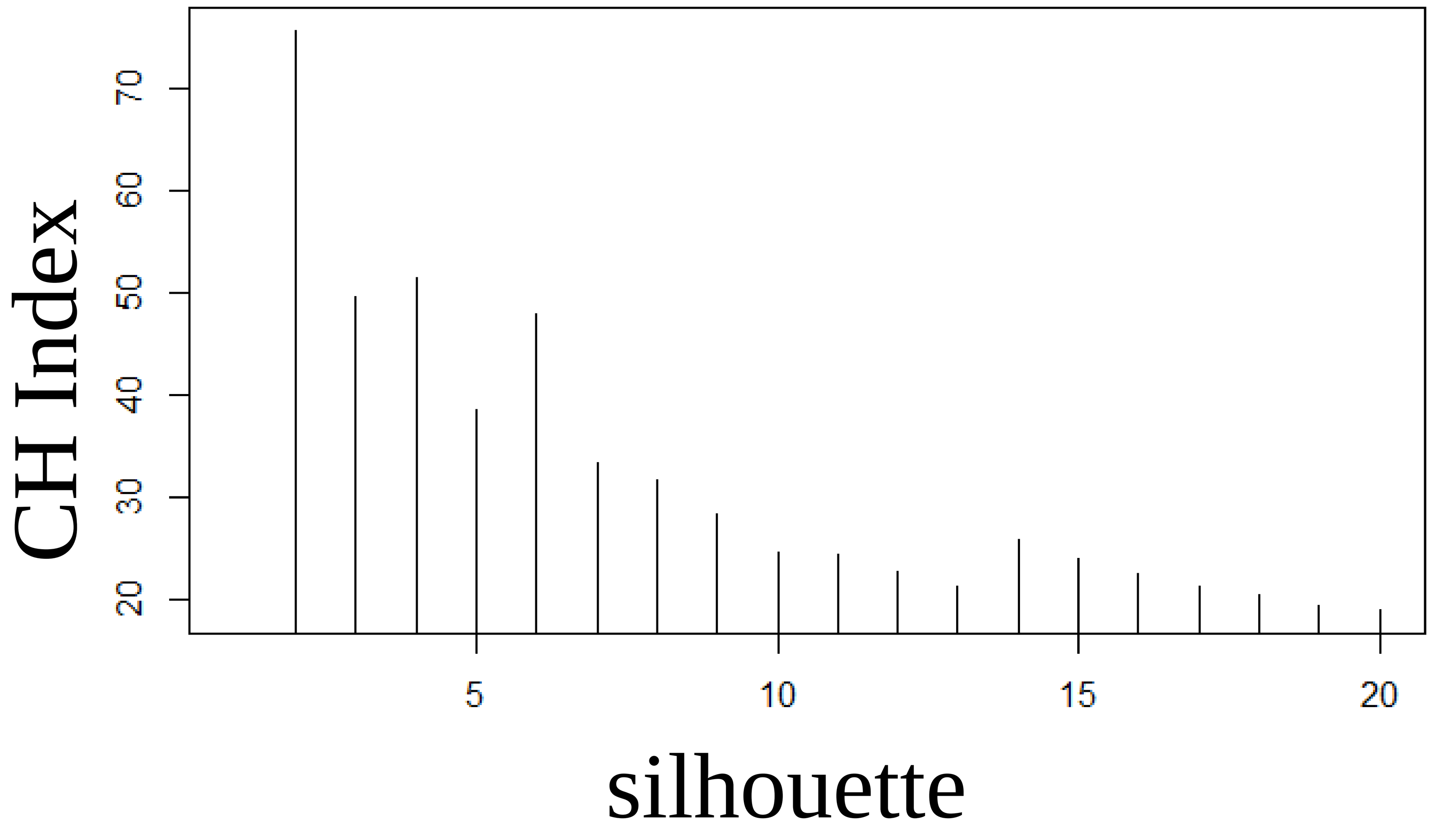

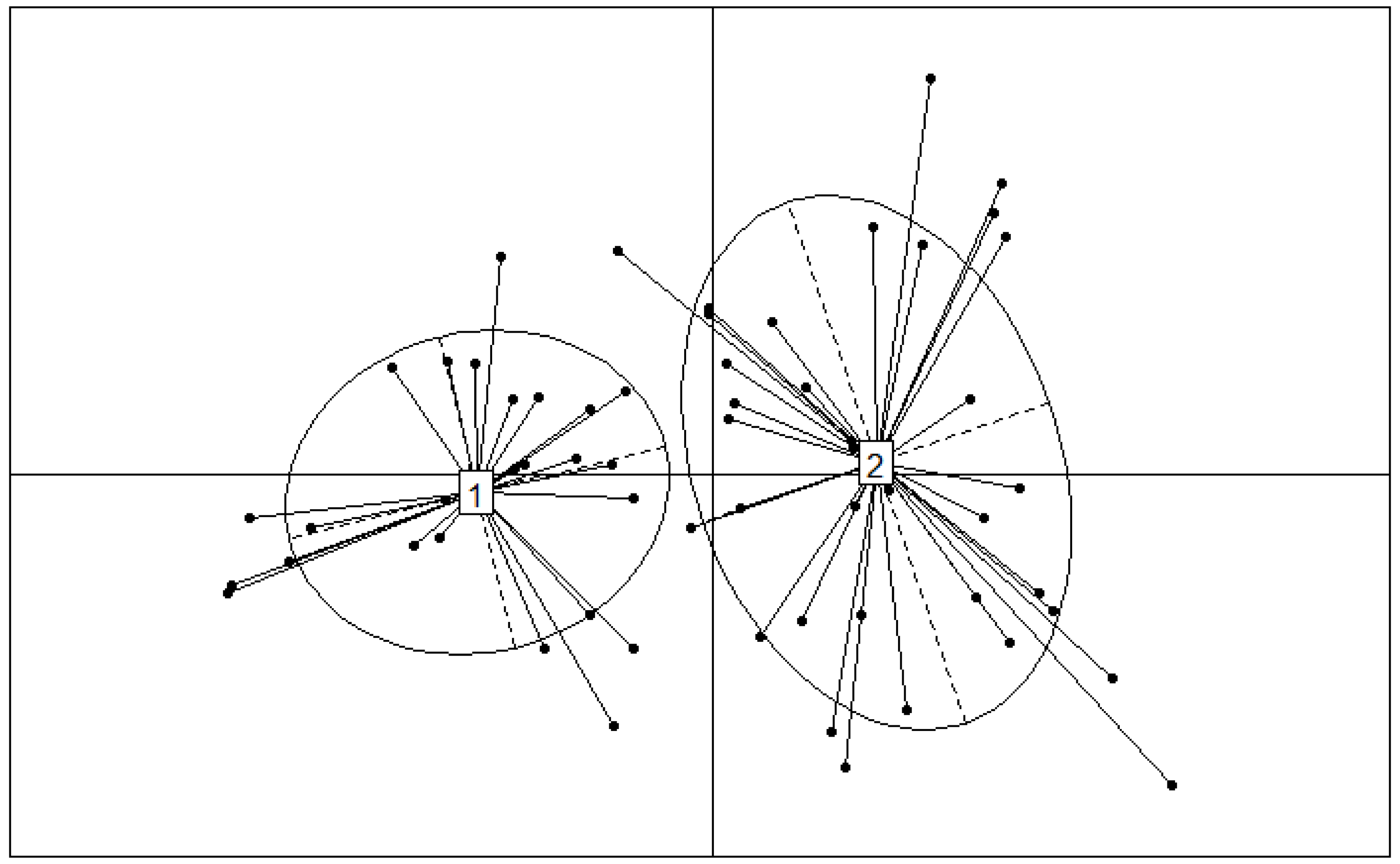

Supplementary Figure S3e

154 days of age

Dataset randomized

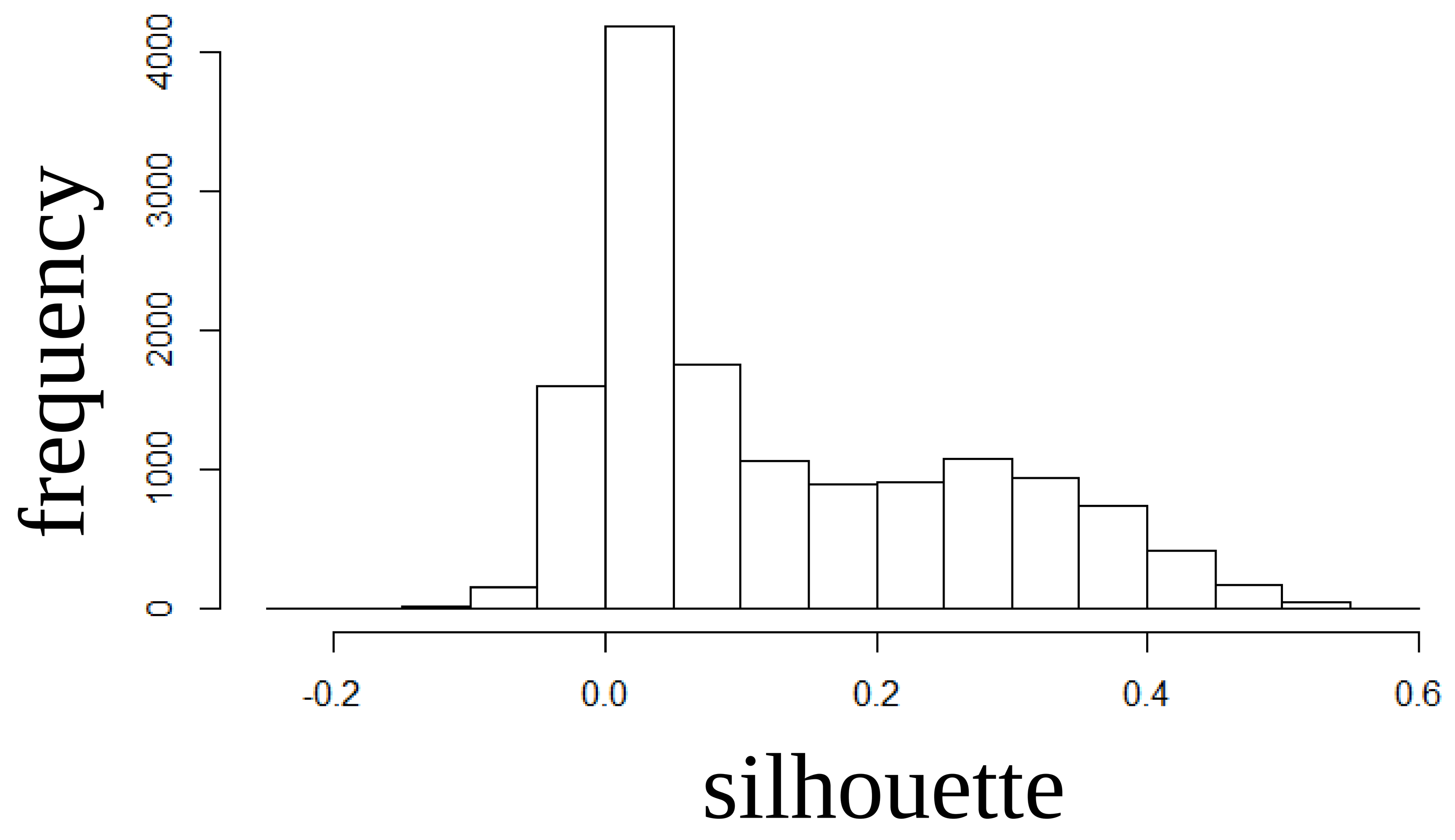

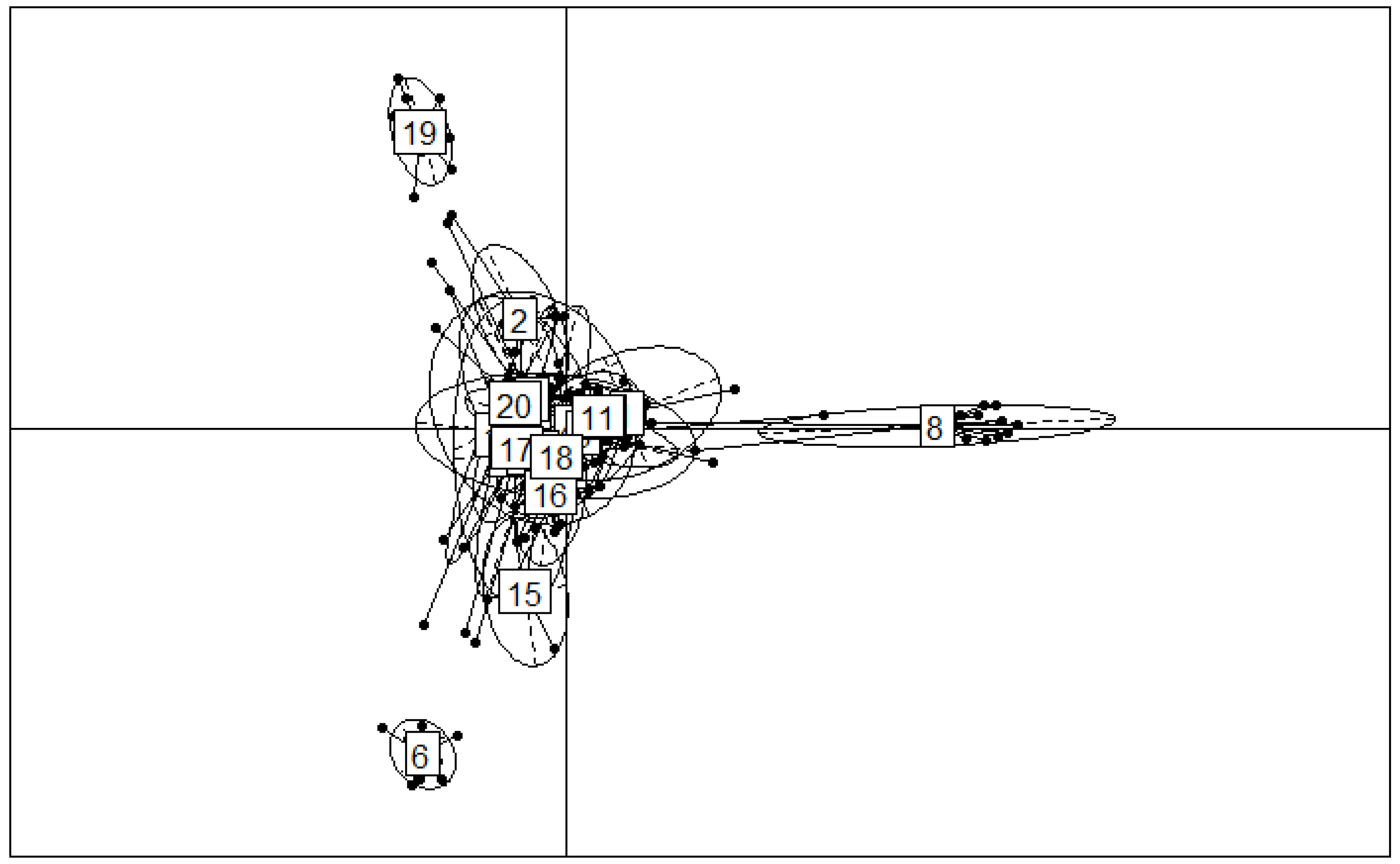

Real dataset

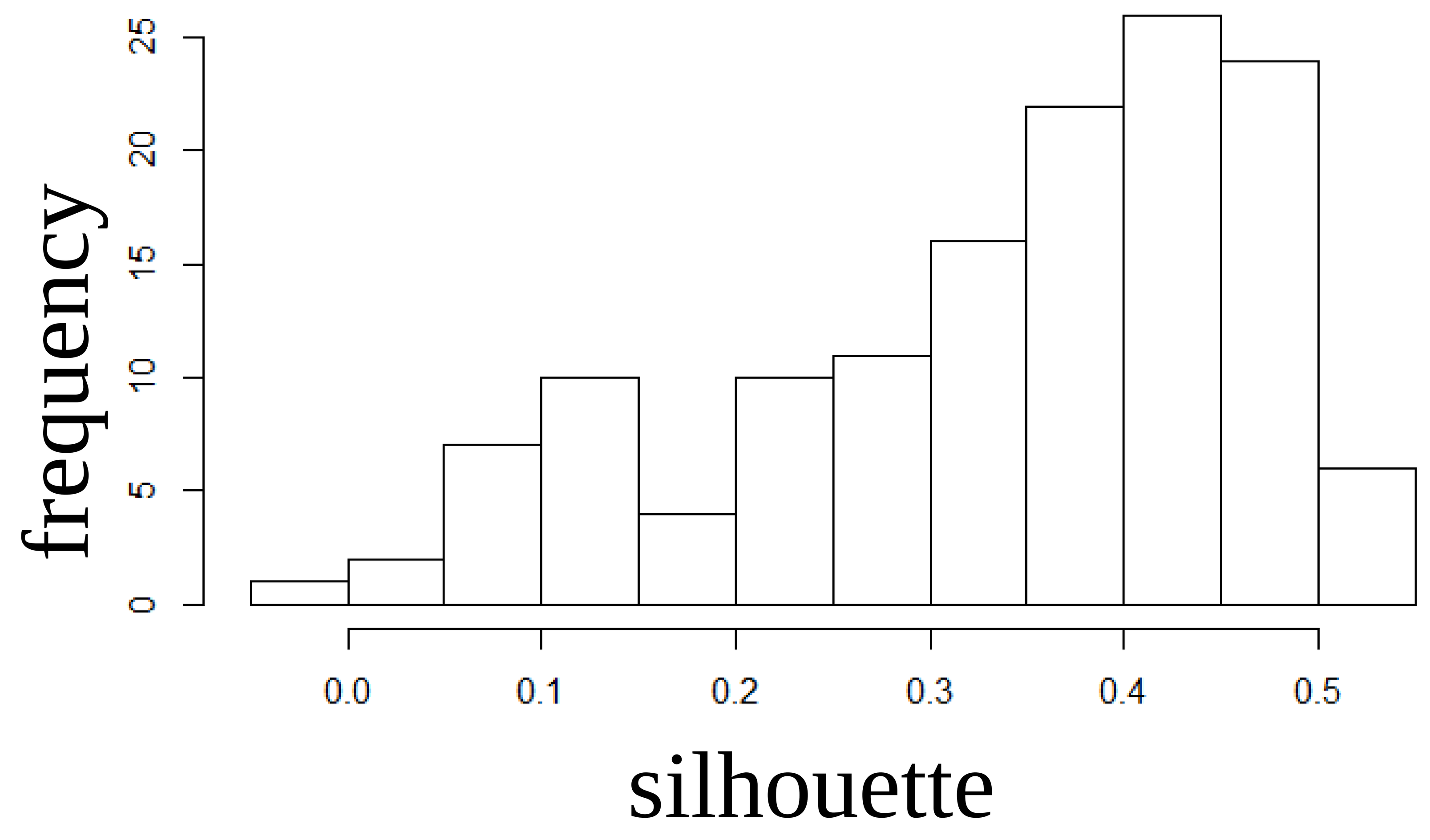

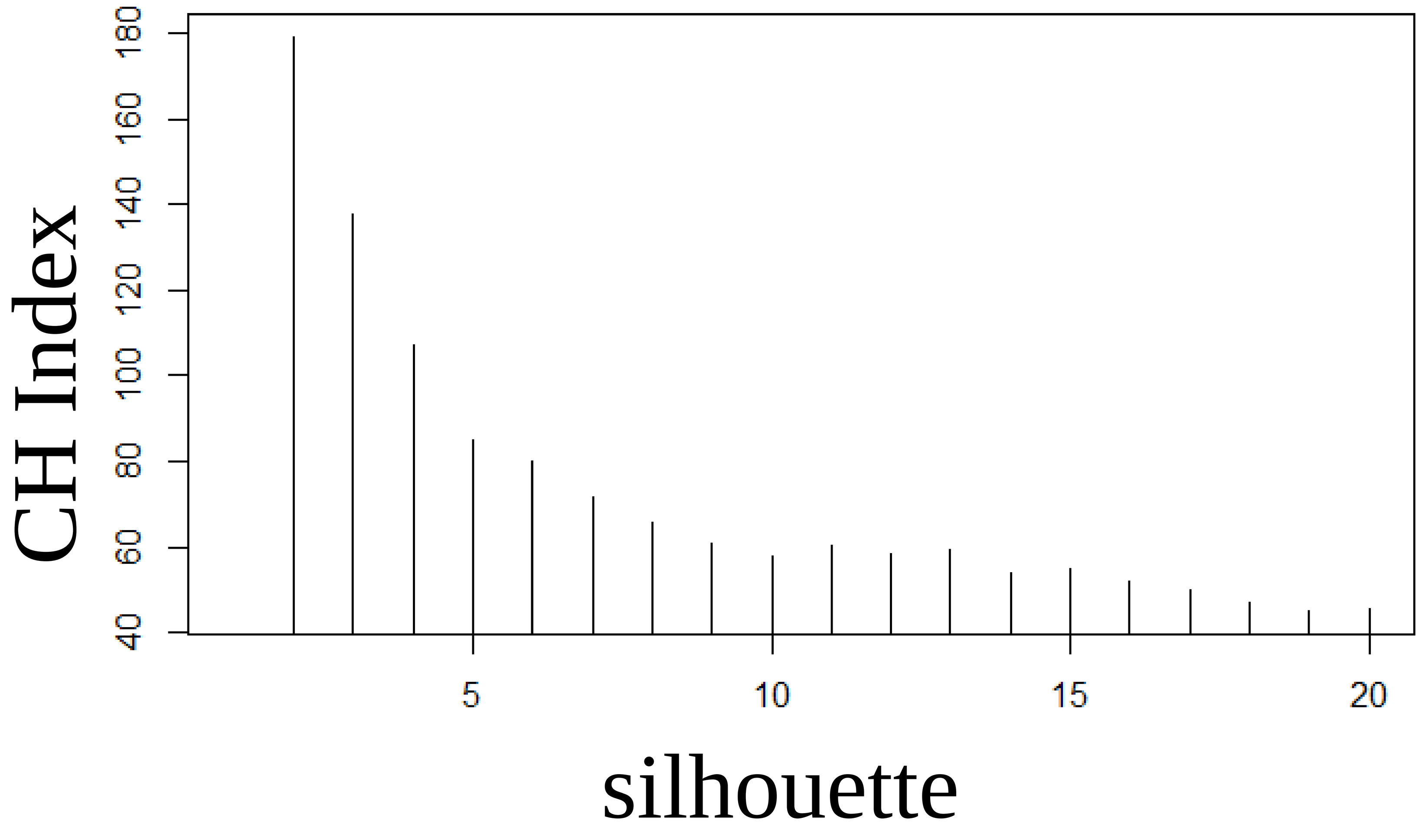

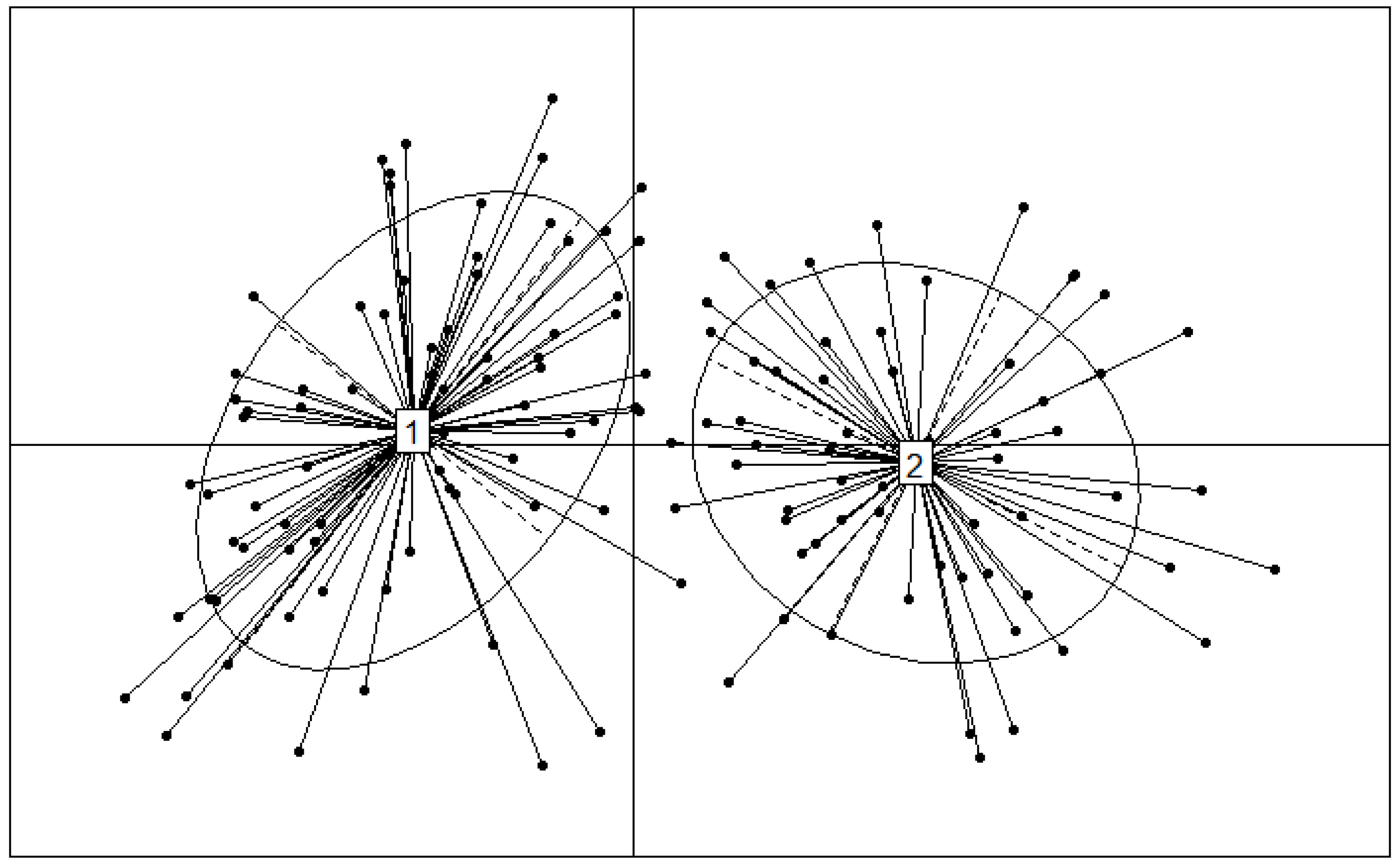

Supplement: Supplementary file 1 [file microorganisms-07-00622-s001.zip › Figure_S4.pdf]
