# Supplementary material for: Longitudinal Analysis of the Microbiota Composition and Enterotypes of Pigs from Post-Weaning to Finishing
Source: Microorganisms. 2019 Nov 28;7(12):622. doi: 10.3390/microorganisms7120622 (PMC6956163; doi:10.3390/microorganisms7120622)
Supplement: Supplementary file 1 [file microorganisms-07-00622-s001.zip › Figure_S5.pdf]

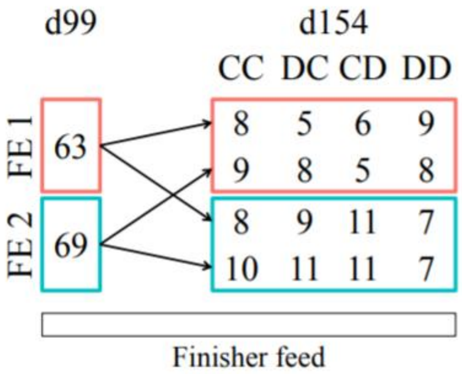

	CC_Group	DC_Group	CD_Group	DD_Group
Pigs in finishing stage enterotype FE1 at Day 154	17	13	11	17
Pigs in finishing stage enterotype FE2 at Day 154	18	20	22	14
Total number of pigs in the group	35	33	33	31
ratio of pigs in FE1	0.48	0.39	0.33	0.54
minimum ratio of FE1 (95% confidence by binomial law)	0.31	0.22	0.18	0.36
max ratio of FE1 (95% confidence by binomial law)	0.66	0.57	0.51	0.72

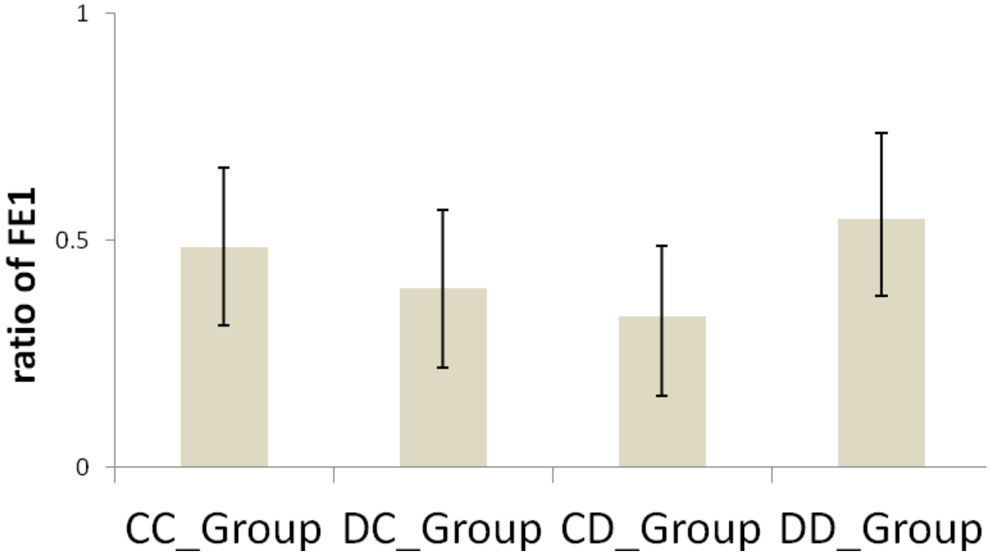

Figure S5: Absence of long-term impact of the DON exposure. Distribution of the pigs in the FE1 and FE2 enterotypes at Day 154 (TOP), calculus of the confidence interval (MIDDLE) and the ratio of FE1 with the 95% confidence interval according to the binomial confidence interval (BOTTOM). The CC, DC, CD and DD experimental groups corresponds to pigs fed with a control finisher diet, exposed to a DON-contaminated diet between 113 d and 119 d, between 134 d et 140 d, and both between 113 d and 119 d, and 134 d and 140 d of age, respectively.

The FE1 and FE2 enterotypes from the finishing stage were equally represented in each group, illustrating the lack of effect of the DON exposure.
