# Supplementary material for: Longitudinal Analysis of the Microbiota Composition and Enterotypes of Pigs from Post-Weaning to Finishing
Source: Microorganisms. 2019 Nov 28;7(12):622. doi: 10.3390/microorganisms7120622 (PMC6956163; doi:10.3390/microorganisms7120622)
Supplement: Supplementary file 1 [file microorganisms-07-00622-s001.zip › Figure_S6.pdf]

Ratio = $\frac{Pathway\_abundance\_PWE1}{Pathway\_abundance\_PWE2}$

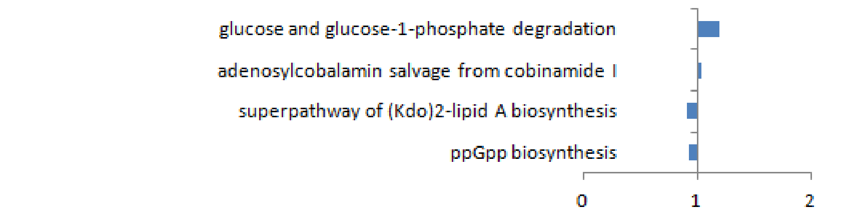

Ratio = $\frac{Pathway\_abundance\_FE1}{Pathway\_abundance\_FE2}$

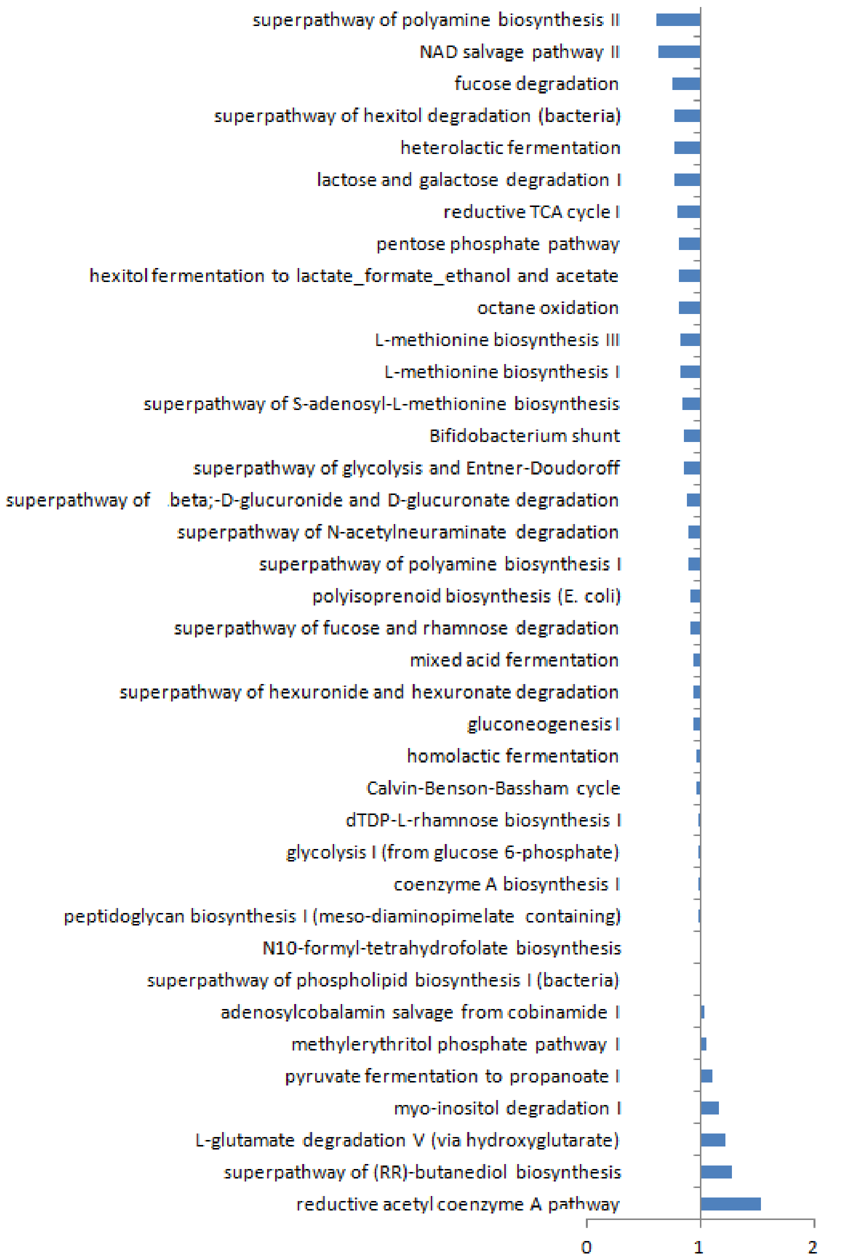

Figure S6: Ratio between the median of pathways that are significantly different between PWE1 ans PWE2 (TOP) and between FE1 and FE2 (BOTTOM). For example, the reductive acetyl coenzyme A pathway is 50% more abundant in FE1 than in FE2 .
