# Supplementary material for: Longitudinal Analysis of the Microbiota Composition and Enterotypes of Pigs from Post-Weaning to Finishing
Source: Microorganisms. 2019 Nov 28;7(12):622. doi: 10.3390/microorganisms7120622 (PMC6956163; doi:10.3390/microorganisms7120622)
Supplement: Supplementary file 1 [file microorganisms-07-00622-s001.zip › Table_S1.docx]

**Table S1**: Composition of experimental growing and finishing diets^1^.

|  | **Growing diet** | **Finishing diets** | |
| --- | --- | --- | --- |
| **Diet** | **Control** | **Control** | **DON contaminated** |
| Ingredients, % as-fed |  |  |  |
| Wheat | 31.91 | - | - |
| Corn | 15.00 | 75.00 | - |
| Corn, DON contaminated | - | - | 75.00 |
| Barley | 25.00 | - | - |
| Wheat bran | 5.00 | 1.54 | 1.64 |
| Rapeseed meal | 7.00 | - | - |
| Soybean meal | 11.50 | 18.10 | 18.00 |
| Vegetable oil | 1.00 | - | - |
| Molasses | - | 2.00 | 2.00 |
| Lysine | 0.74 | - | - |
| Lysine HCL | - | 0.21 | 0.21 |
| DL-Methionine | 0.07 | 0.08 | 0.08 |
| L-Methionine | - | 0.06 | 0.06 |
| Valine | 0.06 | - | - |
| L-Threonin | 0.17 | - | - |
| L-Tryptophan | 0.11 | 0.01 | 0.01 |
| Calcium carbonate | 1.20 | - | - |
| Monocalcium phosphate | 0.19 | - | - |
| Dicalcium phosphate | - | 1.00 | 1.00 |
| Calcium phosphate | - | 1.00 | 1.00 |
| Salt | 0.45 | 0.50 | 0.50 |
| Vitamin-mineral premix^2^ | 0.60 | 0.50 | 0.50 |
|  |  |  |  |
| Analyzed composition^3^ |  |  |  |
| Dry matter, % | 87.8 | 87.0 | 87.0 |
| Organic matter, % | nc^4^ | 79.5 | 77.9 |
| Crude protein, % | 16.5 | 14.2 | 14.1 |
| Crude fat, % | 3.0 | 3.1 | 2.3 |
| Crude fiber, % | 4.0 | 1.5 | 1.7 |
| Neutral detergent fiber, % | 15.2 | 6.3 | 7.5 |
| Acid detergent fiber, % | 5.3 | 1.8 | 2.0 |
| Acid detergent lignin, % | 1.5 | 0.3 | 0.2 |
| Starch, % | 44.0 | 50.6 | 50.7 |
| Gross energy, MJ/kg | 13.4 | 15.8 | 15.7 |
| Net energy, MJ/kg | 9.6 | 10.7 | 10.7 |

^1^ Diet fed in pellet form.

^2^ Proportions for one kilogram of complete diet: vitamin A, 1,000,000 IU; vitamin D, 3, 200,000 IU; vitamin E, 4,000 mg; vitamin B1, 400 mg; vitamin B2, 800 mg; calcium pantothenate, 2,170 mg; niacin, 3,000 mg; vitamin B12, 4 mg; vitamin B6, 200 mg; vitamin K3, 400 mg; folic acid, 200 mg; biotin, 40 mg; choline chloride, 100,000 mg; iron (sulfate), 11,200 mg; iron (carbonate), 4,800 mg; copper (sulfate), 2,000 mg; zinc (oxide), 20,000 mg; manganese (oxide), 8,000 mg; iodine (iodate), 40 mg; cobalt (carbonate), 20 mg; and selenium (selenite), 30 mg.

^3^ As-fed basis. Values were calculated for the same dry matter content (87.0%).

^4^ Not communicated
