# Supplementary material for: Longitudinal Analysis of the Microbiota Composition and Enterotypes of Pigs from Post-Weaning to Finishing
Source: Microorganisms. 2019 Nov 28;7(12):622. doi: 10.3390/microorganisms7120622 (PMC6956163; doi:10.3390/microorganisms7120622)
Supplement: Supplementary file 1 [file microorganisms-07-00622-s001.zip › Table_S2.docx]

**Table S2**: DON challenge effect on female pigs’ fecal microbiota diversity and phyla relative abundance.

|  | **Days of age** | | | | | | |
| --- | --- | --- | --- | --- | --- | --- | --- |
|  | **119d** | | |  | **140d** | | |
| **Item** | **Control** | **Challenged** | **P** |  | **Control** | **Challenged** | **P** |
| Number of pigs^1^ | 28 | 32 |  |  | 30 | 31 |  |
|  |  |  |  |  |  |  |  |
| Diversity indexes |  |  |  |  |  |  |  |
| Nb of OTU | 3,675 | 3,535 | 0.11 |  | 3,759 | 3,680 | 0.31 |
| Shannon index | 7.15 | 7.15 | 0.91 |  | 7.22 | 7.17 | 0.09 |
|  |  |  |  |  |  |  |  |
| Phyla relative abundance (%) |  |  |  |  |  |  |  |
| Firmicutes | 79.48 | 76.99 | 0.07 |  | 78.12 | 78.75 | 0.95 |
| Bacteroidetes | 15.47 | 16.80 | 0.35 |  | 16.19 | 14.18 | 0.20 |
| Spirochaetes | 0.42 | 0.52 | 0.24 |  | 0.62 | 0.77 | 0.06 |
| Actinobacteria | 0.30 | 0.23 | 0.58 |  | 0.17 | 0.22 | <0.01 |
| Proteobacteria | 0.47 | 0.42 | 0.29 |  | 0.46 | 0.33 | 0.02 |
| Fibrobacteres | <0.1 | <0.1 | 0.98 |  | <0.1 | <0.1 | 0.94 |
| Tenericutes | <0.1 | <0.1 | 0.57 |  | <0.1 | <0.1 | 0.09 |
| Unclassified | 3.85 | 5.00 | 0.02 |  | 4.39 | 5.71 | <0.01 |

^a-d^ Least square means within a row with different superscript significantly differ (P < 0.05) in Kruskal-Wallis tests

^1^At 52 d, pigs’ fecal samples from the second replicate only were collected.
