# Supplementary material for: Longitudinal Analysis of the Microbiota Composition and Enterotypes of Pigs from Post-Weaning to Finishing
Source: Microorganisms. 2019 Nov 28;7(12):622. doi: 10.3390/microorganisms7120622 (PMC6956163; doi:10.3390/microorganisms7120622)
Supplement: Supplementary file 1 [file microorganisms-07-00622-s001.zip › Table_S3.docx]

**Table S3:** P-values from Kruskal-Wallis tests for differences in genus relative abundance between the enterotypes at post-weaning (PWE1 vs PWE2) and at finishing (FE1 vs FE2).

| **Genus** | **PWE1 vs PWE2** |  | **Genus** | **FE1 vs FE2 (99d)** |
| --- | --- | --- | --- | --- |
| Lactobacillus | 3.98E-12 |  | Unclassified | 5.41E-22 |
| Sarcina | 1.09E-06 |  | Lactobacillus | 2.41E-21 |
| Unclassified | 1.21E-06 |  | Turicibacter | 8.59E-10 |
| Dialister | 1.24E-06 |  | Clostridium_sensu_stricto | 6.99E-08 |
| Mitsuokella | 5.53E-04 |  | Sarcina | 5.04E-07 |
| Allisonella | 9.37E-04 |  | Clostridium_XI | 3.17E-06 |
| Blautia | 1.74E-03 |  | Treponema | 3.49E-04 |
| Treponema | 6.59E-03 |  | Corynebacterium | 1.02E-03 |
| Eubacterium | 7.52E-03 |  | Staphylococcus | 3.33E-03 |
| Faecalibacterium | 1.03E-02 |  | Bifidobacterium | 3.60E-02 |
| Prevotella | 1.08E-02 |  | Ruminococcus | 3.62E-02 |
| Collinsella | 1.45E-02 |  | Oscillibacter | 3.79E-02 |
| Clostridium_sensu_stricto | 1.80E-02 |  | Bacteroides | 6.14E-02 |
| Olsenella | 4.15E-02 |  | Coprococcus | 6.84E-02 |
| Roseburia | 5.00E-02 |  | Fibrobacter | 7.03E-02 |
| Fusicatenibacter | 5.30E-02 |  | Clostridium_IV | 7.41E-02 |
| Succinivibrio | 9.14E-02 |  | Dorea | 1.24E-01 |
| Fibrobacter | 1.23E-01 |  | Slackia | 2.30E-01 |
| Streptococcus | 1.23E-01 |  | Blautia | 2.77E-01 |
| Ruminococcus2 | 1.47E-01 |  | Erysipelotrichaceae_incertae_sedis | 3.01E-01 |
| Campylobacter | 1.92E-01 |  | Enterococcus | 3.34E-01 |
| Clostridium_XI | 1.94E-01 |  | Roseburia | 3.39E-01 |
| Megasphaera | 2.15E-01 |  | Lachnospiracea_incertae_sedis | 3.39E-01 |
| Enterococcus | 2.27E-01 |  | Prevotella | 3.49E-01 |
| Clostridium_IV | 2.64E-01 |  | Dialister | 3.96E-01 |
| Erysipelotrichaceae_incertae_sedis | 3.24E-01 |  | Collinsella | 4.11E-01 |
| Slackia | 3.88E-01 |  | Escherichia.Shigella | 4.81E-01 |
| Staphylococcus | 3.89E-01 |  | Desulfovibrio | 5.20E-01 |
| Dorea | 4.61E-01 |  | Succinivibrio | 5.25E-01 |
| Bifidobacterium | 4.75E-01 |  | Megasphaera | 6.31E-01 |
| Lachnospiracea_incertae_sedis | 5.07E-01 |  | Eubacterium | 6.74E-01 |
| Desulfovibrio | 5.53E-01 |  | Ruminococcus2 | 7.21E-01 |
| Turicibacter | 5.78E-01 |  | Campylobacter | 7.65E-01 |
| Ruminococcus | 5.94E-01 |  | Allisonella | 8.36E-01 |
| Bacteroides | 6.68E-01 |  | Fusicatenibacter | 8.40E-01 |
| Escherichia.Shigella | 7.01E-01 |  | Olsenella | 8.94E-01 |
| Oscillibacter | 7.38E-01 |  | Faecalibacterium | 9.24E-01 |
| Corynebacterium | 7.54E-01 |  | Streptococcus | 9.48E-01 |
| Coprococcus | 7.85E-01 |  | Mitsuokella | 9.95E-01 |
