# Supplementary figures and images for: Longitudinal Analysis of the Microbiota Composition and Enterotypes of Pigs from Post-Weaning to Finishing
Source: Microorganisms. 2019 Nov 28;7(12):622. doi: 10.3390/microorganisms7120622 (PMC6956163; doi:10.3390/microorganisms7120622)

In total: 76 ♂ and 67 ♀

Longitudinal analysis of  
microbiota composition

Enterotypes analysis

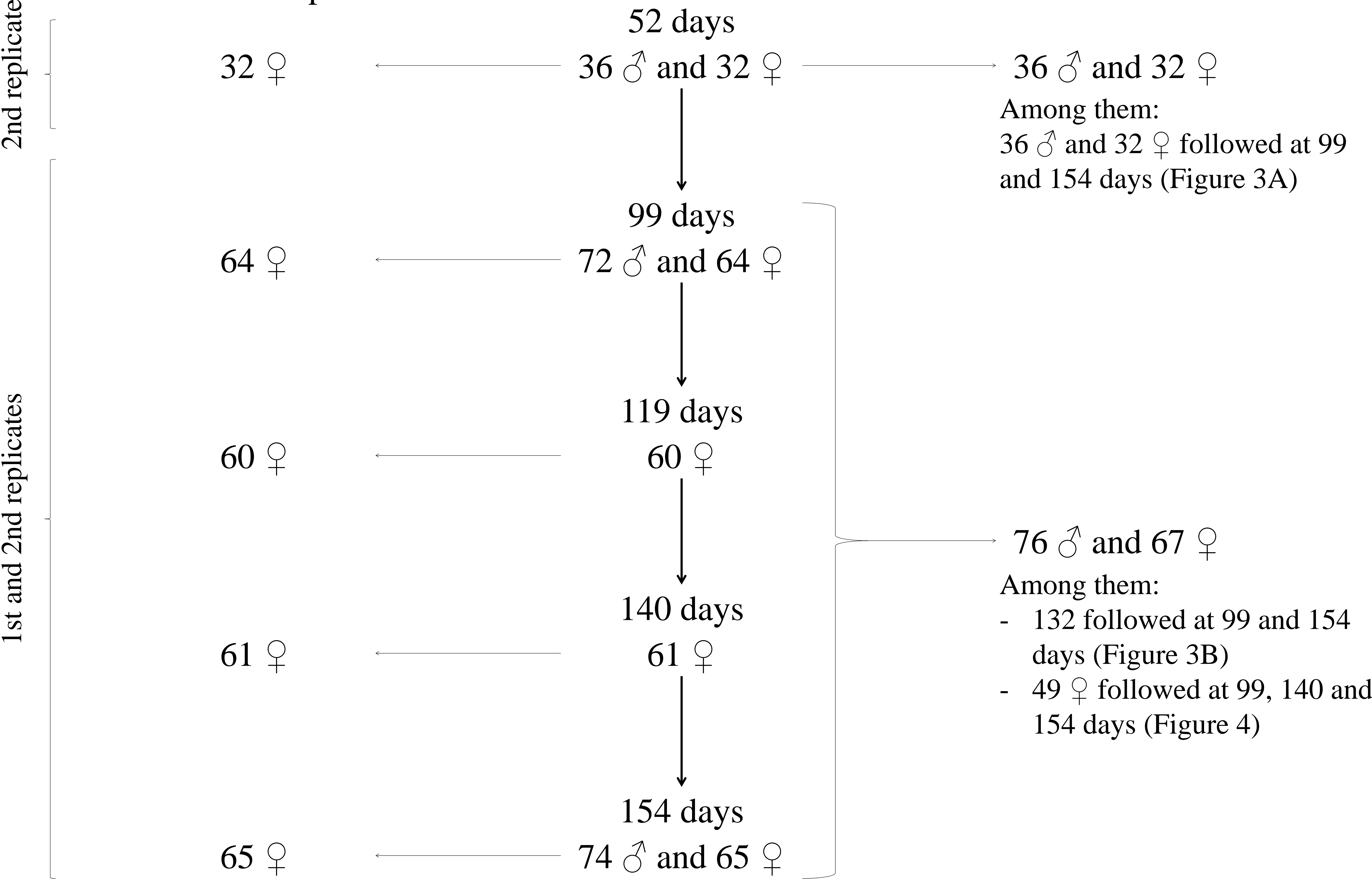

Supplement: Supplementary file 1 [file microorganisms-07-00622-s001.zip › Figure_S2.pdf]

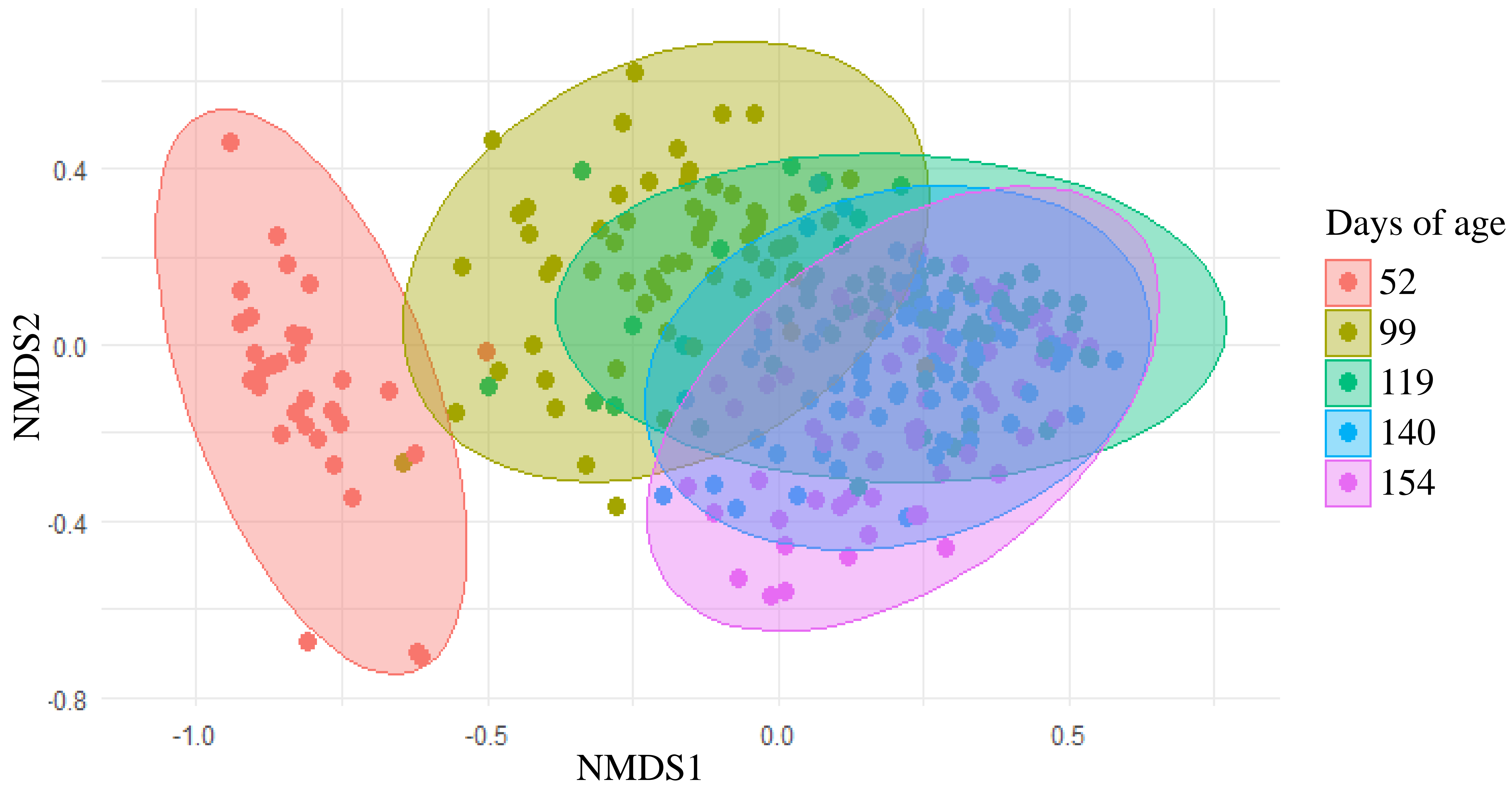

Supplement: Supplementary file 1 [file microorganisms-07-00622-s001.zip › Figure_S3.pdf]
