# Supplementary material for: Longitudinal Analysis of the Microbiota Composition and Enterotypes of Pigs from Post-Weaning to Finishing
Source: Microorganisms. 2019 Nov 28;7(12):622. doi: 10.3390/microorganisms7120622 (PMC6956163; doi:10.3390/microorganisms7120622)
Supplement: Supplementary file 1 [file Figure_S1.pdf › Figure_S1.pdf]

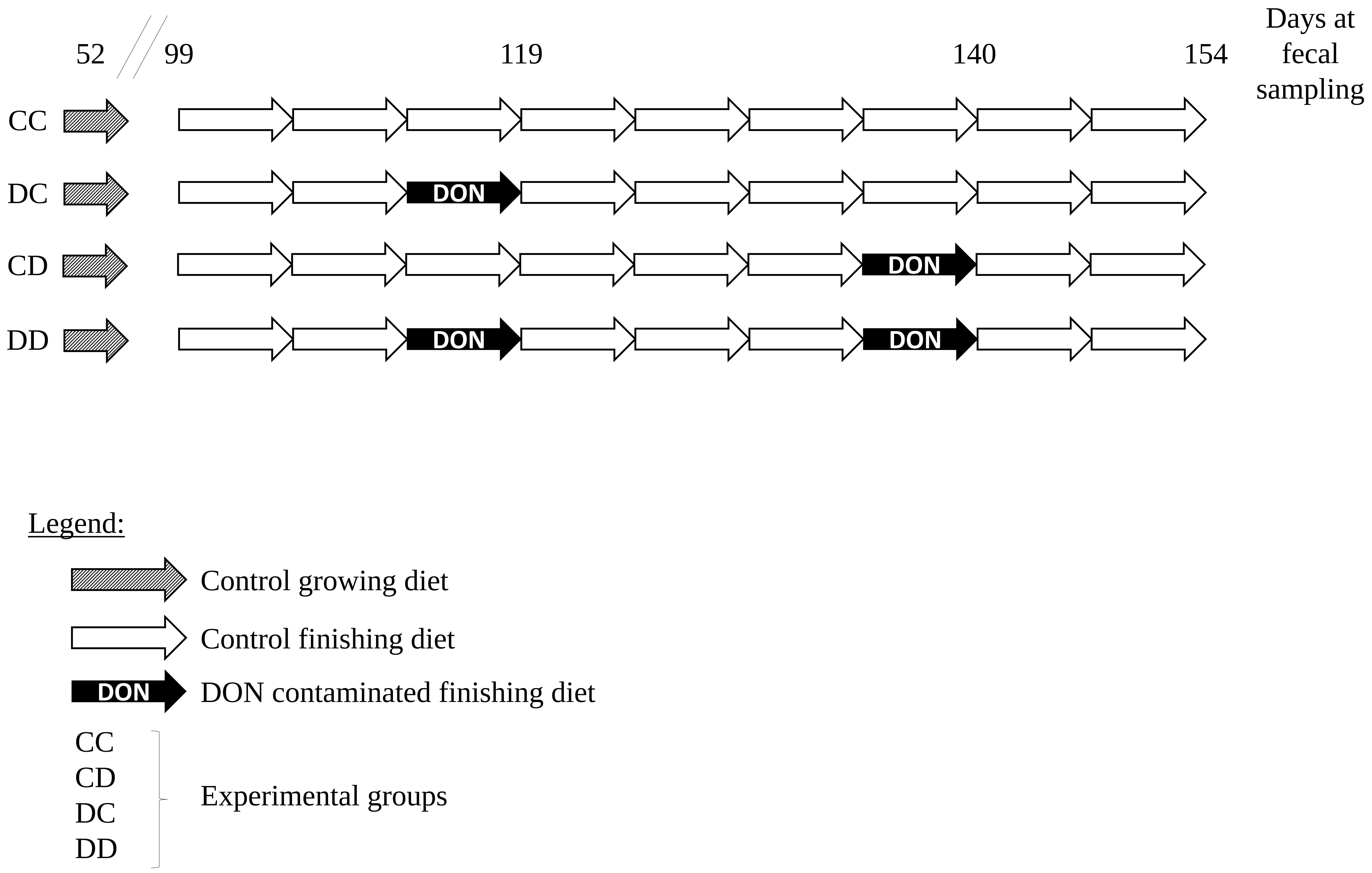
52
99
119
140
154
Days at fecal sampling
CC
DC
CD
DD
DON
DON
DON
DON
Legend:
Control growing diet
Control finishing diet
DON
DON contaminated finishing diet
CC
CD
DC
DD
Experimental groups
