# Supplementary material for: Longitudinal Analysis of the Microbiota Composition and Enterotypes of Pigs from Post-Weaning to Finishing
Source: Microorganisms. 2019 Nov 28;7(12):622. doi: 10.3390/microorganisms7120622 (PMC6956163; doi:10.3390/microorganisms7120622)
Supplement: Supplementary file 1 [file microorganisms-07-00622-s001.zip › Table_S7_pathways_stats.doc]

|  | Pkruskal | Pval(PWE1 vs PWE2) | Pval(FE1 vs FE2) | median in PWE1 | median in PWE2 | median in FE1 | median in FE2 | median in FE3 |
| --- | --- | --- | --- | --- | --- | --- | --- | --- |
| reductive acetyl coenzyme A pathway | 5.82E-19 | 0.5034598 | 0.000846 | 20 | 23 | 60 | 39 | 102 |
| superpathway of (RR)-butanediol biosynthesis | 1.92E-19 | 0.3345941 | 0.006811 | 26 | 27 | 70 | 55 | 137 |
| L-glutamate degradation V (via hydroxyglutarate) | 0.000231154 | 0.1441111 | 0.036272 | 104 | 125 | 172 | 141 | 165 |
| myo-inositol degradation I | 4.12E-11 | 0.944556 | 0.013276 | 25 | 29 | 66 | 57 | 69 |
| pyruvate fermentation to propanoate I | 1.91E-10 | 0.1798685 | 0.004061 | 1716 | 1878 | 2516 | 2285 | 2796 |
| methylerythritol phosphate pathway I | 1.23E-08 | 0.0770651 | 0.034646 | 8836 | 9628 | 10621 | 10164 | 10925 |
| adenosylcobalamin salvage from cobinamide I | 2.59E-08 | 0.0378832 | 0.037883 | 7671 | 8490 | 9031 | 8697 | 8952 |
| superpathway of phospholipid biosynthesis I (bacteria) | 0.009212312 | 0.7279147 | 0.045119 | 14069 | 14044 | 14122 | 14311 | 14225 |
| N10-formyl-tetrahydrofolate biosynthesis | 6.27E-05 | 0.977254 | 0.012465 | 10508 | 10562 | 10718 | 10874 | 10832 |
| peptidoglycan biosynthesis I (meso-diaminopimelate containing) | 5.69E-06 | 0.8273656 | 9.69E-06 | 13133 | 13054 | 13019 | 13260 | 13281 |
| coenzyme A biosynthesis I | 0.00076367 | 0.9908962 | 0.000244 | 12393 | 12386 | 12307 | 12541 | 12437 |
| glycolysis I (from glucose 6-phosphate) | 0.00012561 | 0.9380104 | 3.86E-05 | 13322 | 13349 | 13264 | 13564 | 13454 |
| dTDP-L-rhamnose biosynthesis I | 3.92E-08 | 0.238706 | 5.19E-09 | 11767 | 11593 | 11370 | 11699 | 11344 |
| Calvin-Benson-Bassham cycle | 0.000156221 | 0.264315 | 6.54E-05 | 12816 | 13020 | 12610 | 13050 | 12655 |
| homolactic fermentation | 3.09E-07 | 0.4524899 | 1.71E-07 | 12365 | 12248 | 12046 | 12483 | 11917 |
| gluconeogenesis I | 1.02E-10 | 0.5457307 | 2.49E-09 | 11605 | 11343 | 10556 | 11289 | 11002 |
| superpathway of hexuronide and hexuronate degradation | 0.018623216 | 0.5783237 | 0.018481 | 2344 | 2436 | 2325 | 2493 | 2243 |
| mixed acid fermentation | 1.44E-08 | 0.167115 | 2.85E-06 | 7848 | 7669 | 6968 | 7499 | 7485 |
| superpathway of fucose and rhamnose degradation | 7.84E-05 | 0.2965059 | 0.001078 | 1150 | 1088 | 1004 | 1099 | 964 |
| polyisoprenoid biosynthesis (E. coli) | 1.13E-07 | 0.0979572 | 0.000586 | 9229 | 8241 | 7544 | 8382 | 8495 |
| superpathway of polyamine biosynthesis I | 5.85E-06 | 0.1874942 | 0.017324 | 2339 | 2196 | 2091 | 2336 | 2735 |
| superpathway of N-acetylneuraminate degradation | 4.27E-05 | 0.658287 | 0.001877 | 5758 | 5918 | 4592 | 5167 | 5732 |
| superpathway of &beta;-D-glucuronide and D-glucuronate degradation | 8.14E-05 | 0.7874619 | 1.77E-05 | 2900 | 2992 | 2682 | 3029 | 2654 |
| superpathway of glycolysis and Entner-Doudoroff | 0.00010062 | 0.975612 | 0.002389 | 4028 | 3521 | 2735 | 3196 | 3772 |
| Bifidobacterium shunt | 6.96E-08 | 0.1306482 | 0.00076 | 8993 | 7821 | 6549 | 7732 | 6736 |
| superpathway of S-adenosyl-L-methionine biosynthesis | 3.25E-06 | 0.7865134 | 0.000209 | 5753 | 5570 | 4368 | 5190 | 5285 |
| L-methionine biosynthesis I | 1.73E-06 | 0.8292448 | 0.000153 | 4199 | 4032 | 3080 | 3720 | 3833 |
| L-methionine biosynthesis III | 7.64E-07 | 0.7992241 | 6.31E-05 | 4367 | 4191 | 3156 | 3841 | 3972 |
| octane oxidation | 6.61E-08 | 0.1800464 | 0.021199 | 130 | 87 | 68 | 83 | 24 |
| hexitol fermentation to lactate_formate_ethanol and acetate | 1.39E-09 | 0.6943711 | 2.59E-05 | 2648 | 2570 | 1651 | 2042 | 2786 |
| pentose phosphate pathway | 0.000222292 | 0.3008027 | 0.006687 | 5643 | 4146 | 3555 | 4407 | 4322 |
| reductive TCA cycle I | 1.89E-06 | 0.7852307 | 1.19E-05 | 2208 | 2209 | 1826 | 2299 | 2528 |
| lactose and galactose degradation I | 6.18E-11 | 0.0962664 | 3.84E-05 | 3838 | 3463 | 2128 | 2791 | 2480 |
| heterolactic fermentation | 9.08E-05 | 0.2823704 | 0.008732 | 5378 | 3907 | 3128 | 4109 | 4033 |
| superpathway of hexitol degradation (bacteria) | 1.07E-12 | 0.7414199 | 1.45E-07 | 3613 | 3729 | 2331 | 3073 | 3649 |
| fucose degradation | 3.76E-09 | 0.5902165 | 7.93E-05 | 1143 | 1142 | 785 | 1054 | 639 |
| NAD salvage pathway II | 8.52E-12 | 0.3868187 | 6.30E-07 | 271 | 248 | 133 | 210 | 126 |
| superpathway of polyamine biosynthesis II | 6.98E-09 | 0.8882789 | 0.002255 | 1181 | 1261 | 622 | 1016 | 338 |
| catechol degradation to beta;-ketoadipate | 7.50E-06 | 0.6057167 | 0.029614 | 0 | 0 | 0 | 0 | 0 |
| adenosylcobalamin biosynthesis II (late cobalt incorporation) | 0.008389293 | 0.6250323 | 0.028437 | 0 | 0 | 0 | 0 | 0 |
| superpathway of L-lysine_L-threonine and L-methionine biosynthesis I | 0.017133172 | 0.9905025 | 0.053171 | 7692 | 7833 | 7291 | 7722 | 8196 |
| superpathway of L-aspartate and L-asparagine biosynthesis | 1.85E-06 | 0.5274779 | 0.059971 | 11117 | 11566 | 12059 | 11746 | 12007 |
| fatty acid elongation -- saturated | 0.004478047 | 0.9772519 | 0.063569 | 6619 | 6564 | 5835 | 6110 | 6984 |
| glucose and glucose-1-phosphate degradation | 0.000521642 | 0.0490511 | 0.078135 | 16 | 24 | 22 | 19 | 40 |
| L-leucine degradation I | 7.38E-09 | 0.340805 | 0.089393 | 154 | 92 | 244 | 201 | 441 |
| glycolysis III (from glucose) | 4.28E-05 | 0.7165433 | 0.093044 | 14680 | 14613 | 15015 | 15180 | 15246 |
| D-galacturonate degradation I | 0.057001911 | 0.9084794 | 0.109422 | 3144 | 3031 | 3175 | 3343 | 3413 |
| 3-phenylpropanoate degradation | 0.000798307 | 0.8720226 | 0.110481 | 0 | 0 | 0 | 0 | 0 |
| pyruvate fermentation to butanoate | 2.02E-09 | 0.5899096 | 0.131614 | 383 | 237 | 613 | 509 | 939 |
| L-histidine degradation I | 2.34E-07 | 0.652368 | 0.149998 | 1257 | 1257 | 1725 | 1595 | 1902 |
| superpathway of arginine and polyamine biosynthesis | 0.051064695 | 0.6684018 | 0.155624 | 2898 | 2800 | 2841 | 2952 | 3025 |
| superpathway of glycerol degradation to 13-propanediol | 0.001623607 | 0.7516731 | 0.164901 | 678 | 732 | 629 | 550 | 730 |
| fatty acid &beta;-oxidation I | 3.62E-09 | 0.6417553 | 0.167257 | 191 | 116 | 301 | 257 | 475 |
| D-glucarate degradation I | 0.000232812 | 0.1851701 | 0.18517 | 88 | 73 | 67 | 71 | 79 |
| purine nucleobases degradation I (anaerobic) | 6.24E-07 | 0.6665054 | 0.218585 | 2647 | 2752 | 3090 | 2943 | 3315 |
| pentose phosphate pathway (non-oxidative branch) | 0.006507809 | 0.5541188 | 0.254278 | 17518 | 17601 | 18181 | 18220 | 18503 |
| L-lysine fermentation to acetate and butanoate | 2.26E-08 | 0.5968065 | 0.286668 | 260 | 250 | 308 | 322 | 429 |
| phosphopantothenate biosynthesis I | 2.27E-05 | 0.5926619 | 0.308824 | 7383 | 7486 | 8264 | 8029 | 8480 |
| incomplete reductive TCA cycle | 0.012506606 | 0.4836663 | 0.321078 | 5301 | 4855 | 5276 | 5350 | 6127 |
| methanogenesis from acetate | 0.000553097 | 0.8084705 | 0.332104 | 3050 | 3217 | 3118 | 3112 | 2495 |
| protocatechuate degradation II (ortho-cleavage pathway) | 3.75E-05 | 0.5413697 | 0.336907 | 1 | 0 | 2 | 3 | 5 |
| L-lysine biosynthesis I | 2.36E-08 | 0.0751315 | 0.341114 | 9717 | 10320 | 10814 | 10527 | 11282 |
| ketogluconate metabolism | 3.25E-07 | 0.3478955 | 0.347896 | 0 | 0 | 0 | 0 | 1 |
| aerobactin biosynthesis | 3.30E-07 | 0.3564995 | 0.3565 | 0 | 0 | 0 | 0 | 0 |
| heme biosynthesis II (anaerobic) | 0.001743704 | 0.6924282 | 0.365153 | 1833 | 1573 | 1273 | 1348 | 2108 |
| superpathway of lipopolysaccharide biosynthesis | 3.50E-07 | 0.3696587 | 0.369659 | 1 | 0 | 0 | 0 | 2 |
| 3-phenylpropanoate and 3-(3-hydroxyphenyl)propanoate degradation to 2-oxopent-4-enoate | 4.73E-07 | 0.3706313 | 0.370631 | 0 | 0 | 0 | 0 | 0 |
| L-arginine biosynthesis II (acetyl cycle) | 0.000886968 | 0.3210354 | 0.389958 | 9553 | 9910 | 10369 | 10347 | 10765 |
| biotin biosynthesis I | 5.73E-05 | 0.3975526 | 0.397553 | 976 | 990 | 1245 | 1197 | 1457 |
| O-antigen building blocks biosynthesis (E. coli) | 0.142651772 | 0.9536031 | 0.397715 | 10425 | 10346 | 10392 | 10526 | 10678 |
| L-arginine biosynthesis I (via L-ornithine) | 0.000908089 | 0.2783424 | 0.397934 | 9519 | 9821 | 10223 | 10202 | 10603 |
| acetylene degradation | 0.028372588 | 0.4344414 | 0.422483 | 12261 | 12040 | 11689 | 12015 | 11477 |
| glycogen biosynthesis I (from ADP-D-Glucose) | 0.000499399 | 0.2299416 | 0.423066 | 12362 | 13029 | 13409 | 13452 | 13880 |
| NAD biosynthesis II (from tryptophan) | 2.12E-05 | 0.3332105 | 0.44478 | 0 | 0 | 0 | 0 | 7 |
| superpathway of glycol metabolism and degradation | 8.63E-09 | 0.3368477 | 0.454285 | 14 | 9 | 19 | 15 | 57 |
| pantothenate and coenzyme A biosynthesis I | 0.000564051 | 0.5797359 | 0.454761 | 8726 | 8838 | 9386 | 9154 | 9509 |
| superpathway of histidine_purine_and pyrimidine biosynthesis | 9.92E-07 | 0.4527456 | 0.456969 | 1916 | 1355 | 2224 | 2144 | 3495 |
| L-histidine biosynthesis | 0.031858177 | 0.4620296 | 0.46203 | 9071 | 9314 | 10224 | 10056 | 10176 |
| formaldehyde assimilation I (serine pathway) | 0.004827086 | 0.2102845 | 0.47081 | 5 | 0 | 0 | 3 | 0 |
| colanic acid building blocks biosynthesis | 0.157248968 | 0.9554406 | 0.473491 | 3727 | 3703 | 3938 | 4026 | 3981 |
| L-ornithine biosynthesis | 0.002194524 | 0.4776574 | 0.49931 | 7876 | 8115 | 8889 | 8842 | 9288 |
| TCA cycle IV (2-oxoglutarate decarboxylase) | 2.63E-09 | 0.4640042 | 0.509652 | 59 | 38 | 90 | 84 | 317 |
| superpathway of taurine degradation | 0.068212986 | 0.3115183 | 0.510532 | 5 | 2 | 1 | 2 | 0 |
| glyoxylate cycle | 6.30E-08 | 0.3432683 | 0.522007 | 19 | 12 | 23 | 22 | 87 |
| coenzyme M biosynthesis I | 9.04E-11 | 0.5484944 | 0.550248 | 0 | 0 | 4 | 2 | 4 |
| arginine_ornithine and proline interconversion | 0.075330089 | 0.5623085 | 0.552483 | 1515 | 1537 | 1556 | 1504 | 1727 |
| superpathway of N-acetylglucosamine_N-acetylmannosamine and N-acetylneuraminate degradation | 0.243558281 | 0.6095514 | 0.609551 | 3960 | 4169 | 4154 | 4164 | 4227 |
| heme biosynthesis I (aerobic) | 0.013538765 | 0.5201183 | 0.619358 | 1114 | 904 | 885 | 868 | 1113 |
| lipid IVA biosynthesis | 0.354374589 | 0.9847684 | 0.631948 | 1696 | 1644 | 1826 | 1846 | 1869 |
| superpathway of branched amino acid biosynthesis | 0.000995869 | 0.4173811 | 0.644896 | 11332 | 11698 | 13051 | 12709 | 13159 |
| superpathway of glycolysis_pyruvate dehydrogenase_TCA_and glyoxylate bypass | 6.66E-09 | 0.3053634 | 0.645392 | 66 | 44 | 74 | 67 | 239 |
| superpathway of L-arginine_putrescine_and 4-aminobutanoate degradation | 7.11E-07 | 0.6696344 | 0.669634 | 5 | 4 | 5 | 4 | 44 |
| superpathway of L-arginine and L-ornithine degradation | 7.11E-07 | 0.6696344 | 0.669634 | 5 | 4 | 5 | 4 | 44 |
| L-arginine degradation II (AST pathway) | 7.84E-07 | 0.6503109 | 0.670689 | 3 | 2 | 2 | 2 | 19 |
| superpathway of ornithine degradation | 7.49E-07 | 0.6722667 | 0.672267 | 5 | 3 | 5 | 4 | 38 |
| glycogen degradation I (bacterial) | 0.010864186 | 0.3440309 | 0.689846 | 13527 | 13960 | 14387 | 14318 | 14552 |
| enterobacterial common antigen biosynthesis | 5.49E-07 | 0.6939217 | 0.693922 | 6 | 4 | 6 | 4 | 40 |
| enterobactin biosynthesis | 6.44E-07 | 0.7005961 | 0.700596 | 12 | 9 | 13 | 10 | 102 |
| superpathway of chorismate metabolism | 3.02E-07 | 0.7051891 | 0.705189 | 53 | 41 | 50 | 42 | 156 |
| superpathway of methylglyoxal degradation | 5.05E-07 | 0.7107403 | 0.71074 | 5 | 3 | 5 | 3 | 30 |
| CMP-3-deoxy-D-manno-octulosonate biosynthesis I | 0.732273526 | 0.8809138 | 0.723937 | 1709 | 1786 | 1740 | 1811 | 1770 |
| superpathway of purine nucleotides de novo biosynthesis II | 0.027129479 | 0.7357724 | 0.735772 | 10334 | 10299 | 10901 | 10746 | 10962 |
| superpathway of aromatic amino acid biosynthesis | 0.005737242 | 0.3835828 | 0.785153 | 11605 | 12027 | 12970 | 12695 | 13001 |
| superpathway of fatty acid biosynthesis initiation (E. coli) | 0.628754267 | 0.832629 | 0.832629 | 2374 | 2116 | 2217 | 2100 | 2186 |
| glycolysis V (Pyrococcus) | 0.628789924 | 0.840425 | 0.840425 | 10 | 7 | 10 | 7 | 7 |
| D-galactarate degradation I | 0.735172 | 0.8713788 | 0.871379 | 6 | 4 | 6 | 6 | 4 |
| superpathway of D-glucarate and D-galactarate degradation | 0.735172 | 0.8713788 | 0.871379 | 6 | 4 | 6 | 6 | 4 |
| ppGpp biosynthesis | 0.00014209 | 0.0323914 | 0.88273 | 43 | 68 | 34 | 37 | 71 |
| chorismate biosynthesis I | 0.01219862 | 0.4052058 | 0.883001 | 11143 | 11408 | 12301 | 12159 | 12417 |
| superpathway of (Kdo)2-lipid A biosynthesis | 0.000217075 | 0.0345672 | 0.897797 | 130 | 191 | 101 | 111 | 211 |
| superpathway of tetrahydrofolate biosynthesis and salvage | 0.009081952 | 0.9369149 | 0.939149 | 10126 | 10111 | 10674 | 10578 | 10763 |
| L-isoleucine biosynthesis I (from threonine) | 0.002238254 | 0.4067224 | 0.989257 | 11829 | 12328 | 13524 | 13329 | 13636 |
